# *Poria cocos* compounds targeting neuropeptide Y1 receptor (Y_1_R) for weight management: A computational ligand- and structure-based study with molecular dynamics simulations identified beta-amyrin acetate as a putative Y_1_R inhibitor

**DOI:** 10.1371/journal.pone.0277873

**Published:** 2023-06-30

**Authors:** Ann Rann Wong, Andrew Hung, Angela Wei Hong Yang, Harsharn Gill, George Binh Lenon

**Affiliations:** 1 School of Health and Biomedical Sciences, RMIT University, Bundoora, Victoria, Australia; 2 School of Science, RMIT University, Melbourne, Victoria, Australia; Alagappa University, INDIA

## Abstract

*Poria cocos* (PC) is a medicinal herb frequently used in weight-loss clinical trials, however the mechanisms by which its compounds target orexigenic receptors including the neuropeptide Y_1_ receptor (Y_1_R) remain largely unknown. This study aimed to screen PC compounds for favourable pharmacokinetics profiles and examine their molecular mechanisms targeting Y_1_R. Forty-three PC compounds were systematically sought from pharmacological databases and docked with Y_1_R (PDB: 5ZBQ). By comparing the relative binding affinities, pharmacokinetics and toxicity profiles, we hypothesised that compounds designated **PC1** 3,4-Dihydroxybenzoic acid, **PC8** Vanillic acid, **PC40** 1-(alpha-L-Ribofuranosyl)uracil, could be potential antagonists as they contact major residues Asn283 and Asp287, similar to various potent Y_1_R antagonists. In addition, **PC21** Poricoic acid B, **PC22** Poricoic acid G and **PC43** 16alpha,25-Dihydroxy-24-methylene-3,4-secolanosta-4(28),7,9(11)-triene-3,21-dioic acid, contacting Asn299, Asp104 and Asp200 proximal to the extracellular surface could also interfere with agonist binding by stabilising the extracellular loop (ECL) 2 of Y_1_R in a closed position. Owing to their selective interaction with Phe302, an important residue in binding of selective Y_1_R antagonists, **PC12** beta-Amyrin acetate, **PC26** 3-Epidehydrotumulosic acid and **PC27** Cerevisterol were proposed as putative antagonists. Following the consensus approach, **PC12** beta-Amyrin acetate, **PC26** 3-Epidehydrotumulosic acid and **PC27** Cerevisterol were identified as candidate compounds due to their high affinities (-12.2, -11.0 and -10.8 kcal, respectively), high drug-likeness and low toxicity profiles. Trajectory analyses and energy contributions of **PC12**-Y_1_R complex further confirmed their structural stability and favourable binding free energies, highlighting the feasibility and possible development of **PC12** beta-Amyrin acetate as a future Y_1_R inhibitor.

## Introduction

Obesity, characterised by excessive fat accumulation that poses a risk to health, has been a growing health concern as it has been associated with serious and often life-threatening comorbidities. Globally, the prevalence of obesity in adults has increased significantly in the past 4–5 decades, with estimates of 281 and 309 million men and women identified with obesity in 2016 [1]. Compared to diabetes and other medications related to comorbidities of obesity, the number of anti-obesity pharmacotherapy is relatively limited. Five FDA approved medications for obesity are orlistat, phentermine-topiramate, naltrexone-bupropion, liraglutide and the recently approved semaglutide [2–6]. Except orlistat which works to reduce fat digestion and absorption, four of these medications target the central nervous system to control food intake and minimise binge-eating behaviour. With these approaches, it is not without a risk of adverse events associated with gastrointestinal discomfort, depressive behaviours, and surgical complications [7–9]. Considering the limitations of current approaches, many patients resort to complementary and alternative strategies for obesity management, including Chinese herbal medicine.

*Poria cocos* (PC) (Schw.) Wolf. also known as Fu ling, has been a widely used herb among Chinese herbal formulations for weight management and lipid-modulation. PC is the dried sclerotium of *Wolfiporia cocos*, a saprophytic fungus in the family of Polyporaceae. It is known to be enriched with triterpenes and polysaccharides, with anti-inflammatory, anti-tumour and anti-oxidant properties [10]. Its extensive therapeutic effects have attracted considerable research attention, particularly for the management of metabolic diseases. As observed in multiple systematic reviews and meta-analyses, PC was reported to be one of ten most highly prescribed herbs in obesity clinical trials [11, 12]. Furthermore, for dyslipidaemia studies on humans [13] and ob/ob mice [14, 15], PC has also been a common focus. Although various clinical and experimental studies of PC-containing herbs and formulations have indicated significant improvements in clinical and biochemical outcomes, the molecular mechanisms of PC compounds and their potential as druggable candidates remain largely unknown.

To elucidate the mechanistic action of PC compounds, protein targets were sought from the neuroactive ligand-receptor pathway, one of the most important targets to regulate feeding behaviour and energy homeostasis. Among the neuropeptide Y (NPY) receptors, recent studies have been focusing on the Y1 subtype (Y_1_R) as a therapeutic target for the management of obesity and eating disorders [16, 17]. Belonging to class A (rhodopsin-like) G-protein coupled receptor (GPCR), Y_1_R governs a range of signal transduction and is modulated by a broad range of stimuli included small peptides, lipid analogues, and endogenous and exogenous small molecules. The Y_1_Rs are generally expressed in the basal ganglia and the limbic system, with specific co-localisation in the cerebral cortex, caudate putamen and central amygdala [18]. They have also been found in the subcutaneous and visceral adipose tissues by which they could alter metabolic parameters in visceral obesity [19]. The role of Y_1_R in obesity pathophysiology is primarily induced by a polymorphism in the Y_1_R gene with a Cystosine to Thymidine nucleotide substitution. Clinical studies on 306 obese participants with C to T polymorphism showed a lower tolerance for fasting [19]. Furthermore, the central administrations of antagonistic small molecules such as BIBP3226, LY357897 or 1229U91 have also resulted in a significant reduction in feeding behaviour in rats [20]. Thus, these studies support the role of Y_1_R on obesity development and therefore Y_1_R is selected to be the primary target of this research.

This study aimed to explore the mechanisms of actions of PC compounds targeting Y_1_R and examine their pharmacokinetic profiles as druggable candidates. Computational molecular docking using blind and focused approaches [21] was conducted to predict possible binding sites and to examine the binding affinity and poses of PC compounds on the inactive Y_1_R. The drug-likeness, absorption, distribution, metabolism, excretion, and toxicity (ADMET) profiles were examined using QSAR approaches. By comparing the binding affinities, ligand-residue interaction, pharmacokinetics profile, and molecular simulations of PC compounds against the native co-crystalised ligand, we proposed several putative inhibitors, which could be worthwhile for further *in vivo* and *in vitro* investigations as novel small molecules targeting Y_1_R.

## Materials and methods

### Identification and preparation of PC compounds and Y_1_R target

PC compounds were comprehensively searched on the Traditional Chinese Medicine Information Database (TCM-ID) [22] and the Traditional Chinese Medicine Systems Pharmacology platform (TCMSP) [23] using keywords including ‘*Poria cocos*’, ‘Fu ling’, and ‘茯苓’ up to October 2021. The criteria for screening of duplicated compounds were based on several molecular descriptors, including the PubChem CID, compound name, molecular formula, and molecular weight. The 3D conformer structures of included PC compounds were downloaded in sdf or mol2 format, and were subsequently converted into pdbqt format using Open babel (v 3.1.1) [24] following on the requirements of AutoDock Vina. Duplicates were removed based on similarities in molecular descriptors. Missing bonds and hydrogens were repaired, and all 3D structures were converted into pdbqt format for docking. No limit was set for the number of torsions.

The structure of Y_1_R was obtained from RCSB PDB (PDB: 5ZBQ) [25] and processed in Visual Molecular Dynamics (VMD, v1.9.3) [26]. It adopts an inactive conformation co-crystallised with a known antagonist UR-MK299 at its canonical binding site. It has been reported that UR-MK299 has a PPARγ IC_50_ of 80 nM and K*i* of 28.67nM [27] using a LanthaScreen competitive binding assay and was therefore used as a positive control for this study. The antagonist contains three benzene rings and various carboxyl groups, forming seven hydrogen bonds with Gln219, Asn283 and Asp287 on helices V and VI, by which two of three residues (Asn283 and Asp287) were suggested to be crucial for Y_1_R inhibition [25]. The native ligand (UR-MK299) was isolated from the original PDB structures for re-docking as controls in both blind and focused docking conditions. In the latter case, only the region near the known ligand binding site of Y_1_R was probed. For the preparation of the original Y_1_R PDB, missing loops and side chains were fixed through homology modelling with subsequent energy minimisation on the SWISS-MODEL [28] server based on target sequence obtained from UniProt [25]. The PDB structure was converted into the pdbqt format via Open babel (v 3.1.1) [24] for docking in AutoDock Vina (v1.1.2) [29].

### Physicochemical space examination, drug-likeness prediction, and pharmacokinetics profiling

PC compounds were subjected to quantitative structural-activity analyses (QSAR) using Data Warrior, an open source cheminformatics tool [30] to examine their chemical space and drug-likeness potential. Known Y_1_R ligands were obtained from ChEMBL database [31] and structural variables were used as comparison. Pharmacokinetics profiling including absorption, metabolism, distribution, excretion and toxicity (ADMET) indexes were obtained from running PC compounds through a graph-based structural signature modelling server, pkCSM [32]. These preliminary ligand-based QSAR predictions offer complementary roles to support structural screening such as the molecular docking and molecular dynamics (MD) simulations performed in the next sections.

### Molecular docking and ligand-residue interaction analyses

Computational docking between Y_1_R and PC compounds were first conducted by blind docking to predict available binding sites of Y_1_R. Both allosteric and canonical binding sites were examined. The final binding site selection for focused docking was guided by (a) blind docking outcomes and (b) experimentally known sites [25] to provide more accurate binding poses and energy scores with less computational power [21]. Docking was performed in Autodock Vina [29] with all ligands set to allow full torsional flexibility while proteins were kept in a fixed position. By referencing the co-crystalised ligand coordinates, the grid size and grid center for blind docking were set to 46.69 x 48.12 x 78.56 Å (center *x* = -44.33, *y* = -21.39, *z* = 83.53) and that of focused docking were set to 36.14 x 28.99 x 32.20 Å (center *x* = -48.98, *y* = -20.01, *z* = 68.21). The Intel Xeon Cascade Lake processor nodes of the high-performance computing cluster (‘Gadi’) housed at the National Computational Infrastructure (Canberra, Australia) was used to perform AutoDock Vina calculations. Subsequently, hydrogen and hydrophobic interactions on docked protein-ligand complexes were assessed by the protein-ligand interaction profiler (PLIP) [33] according to pre-specified bond angles and distances cut-offs (S1 Table), and visualised as networks in Gephi (0.9.3) [34].

### Molecular dynamics simulations and free binding energy calculations

To examine the stability of protein-ligand complexes, the candidate PC compounds with the most favourable drug-likeness, ADMET properties, docking energy scores and binding poses were selected for molecular dynamics simulation against the native ligand UR-MK299. Ligand topology and parameter files were generated using CGenFF [35, 36] while the membrane system was prepared using CHARMM36m force field [37]. The system was constructed from CHARMM-GUI membrane builder module using the replacement method [38]. Disulfide bonds between Cys113-Cys198 and Cys33-Cys296 were specified, and the protein orientation was set by outputs from the PPM 2.0 server along the Z-axis. The system consists of POPC components distributed in a 1:1 ratio in the upper (129 molecules) and lower (130 molecules) leaflet, which was solvated with TIP3P water model (25190 molecules) and neutralised with 0.15M of K^+^ and Cl^-^ counterions. Water thickness of at least 22.5 Å on the top and bottom ends was applied to the system box. Energy minimisation was carried out using the steepest descent algorithm following the suggested equilibration scheme to reduce force constraints [38] and periodic boundary conditions were applied to produce constant temperature at 310K with the Nose-Hoover thermostat [39] and constant pressure set at 1 atm using the Parrinello-Rahman barostat using semi-isotropic coupling [40]. Long-range electrostatic forces were calculated from the Particle Mesh Ewald (PME) method [41], and all covalent bonds involving hydrogen atoms were constrained using the LINCS algorithm [42]. The final production runs were performed on GROMACS 2020.4 for 100 ns in triplicates [43]. To examine the stability of ligand binding, the root-mean-squared deviation (RMSD), root-mean-squared fluctuation (RMSF), number of hydrogen bonds and number of contacts were examined. Additionally, binding free energies were calculated by Molecular Mechanics-Poisson Boltzmann Surface Area (MM-PBSA) using the g_mmpbsa tool [44] from the final 1ns of the stabilised trajectory. For these MM-PBSA calculations, the grid spacing was set to 0.05 nm, the solvent dielectric constant was set to 80, and the solute dielectric constant was set to 2. The non-polar energy contribution was approximated based on the solvent-accessible surface area (SASA) model, with a probe radius set to 0.14 nm. The linear Poisson−Boltzmann equation (LPBE) implemented in the Adaptive Poisson-Boltzmann Solver (APBS) package was used. The list of input parameters for MM-PBSA calculations applied in this study was determined following the specification in the original publication of g_mmpbsa tool [44].

## Results and discussion

### Virtual screening through ligand- and structure-based approaches

#### Ligand-based screening and pharmacokinetics profiling revealed a high number of promising drug-like PC compounds occupying similar chemical space as known Y_1_R inhibitors

**Fig 1:**
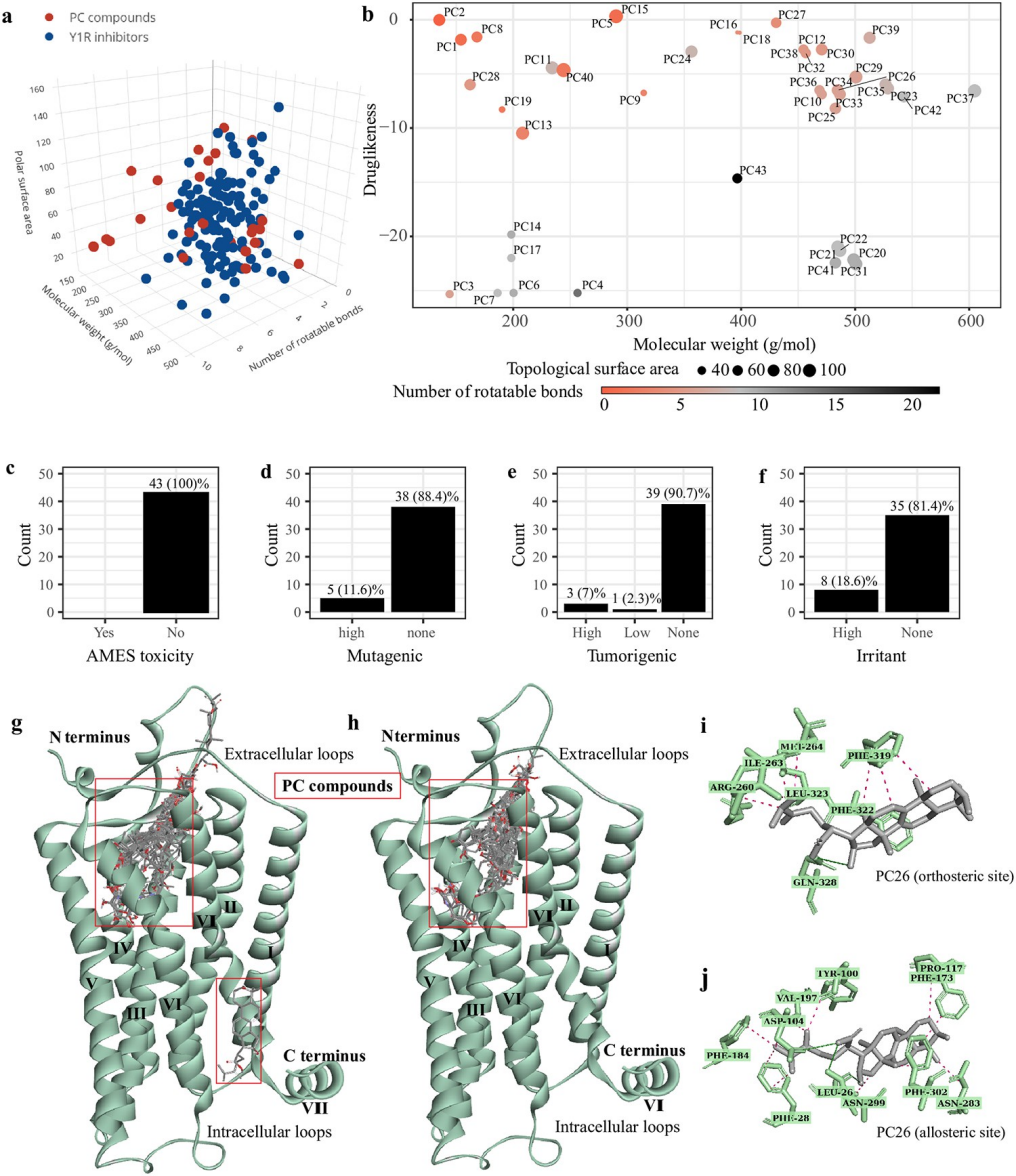
Ligand- and structure-based screening outcomes. (a) Chemical space comparison of PC compounds (red) and known Y_1_R inhibitors (blue). (b) Drug-likeness prediction of PC compounds based on substructure fragments. Toxicity indexes include AMES (c), mutagenicity (d), tumorgenicity (e), and irritant (f) following QSAR analysis from pkCSM and open molecules. 3D ligand-residue interactions in blind (g) and focused (h) docking, with an example compound PC12 at the orthosteric (i) and allosteric (j) sites.

The QSAR modelling of 43 PC compounds (S1 Fig) and 173 known Y_1_R inhibitors suggest that they share similar chemical space characterised by molecular weight, cLogP and topological surface area, particularly for PC compounds with higher molecular weight (Fig 1a). This is not surprising as most therapeutic drug-like molecules range between 350 to 500 g/mol, whereas a molecular weight of < 350 g/mol appears to be more lead-like [45]. Fig 1b shows the drug-likeness scores of PC compounds, and 62.8% of compounds approached a positive score. Interestingly, compounds with a lower rotatable bond count, between 0 to 7, exhibited higher drug-likeness scores. This could be due to their relatively higher oral bioavailability, as previous studies have demonstrated a correlation between the number of rotatable bonds and oral bioavailability, irrespective of molecular weight [46, 47]. The absorption, metabolism, distribution, and excretion (ADMET) indexes were illustrated with detailed cut-off points in S2 Fig. Following pkCSM prediction, most PC compounds exhibit favourable ADMET ranges which suggest that they could be readily bioavailable. According to open molecules and pkCSM analyses, most PC compounds appear to be within safe limits of AMES toxicity, and are generally non mutagenic, carcinogenic or irritative (Fig 1c–1f). Although several compounds had low drug-likeness or unfavourable ADMET properties, possible compound re-design guided by these parameters may help synthesise better molecules for Y_1_R. For example, compounds predicted to have low GI absorption rates could be alternatively administered, via nasal, pulmonary, subcutaneous, or transdermal routes or assisted with micro and nanotechnologies, similar to insulin delivery methods [48]. In addition, nanotechnology such as using nano gold particles have been reported to improve absorption rates of PC compounds [49]. Hence these sub-optimal pharmacokinetics profiles may not necessarily be a major concern for the present study. However, it is important to note that the variability and limitations of machine-learning based predictions exist [50]. Notably, non-important fragments may be more heavily weighted over relevant bioactive fragments involved in binding, and many factors including the quality of input molecules, the choice of descriptors and the selection of statistical modelling and validation methods could significantly alter the accuracy of predictive models. Considering the limitations from ligand-based screening, all PC compounds were subjected to structure-based molecular docking for binding affinity scoring, and a candidate compound is proposed for molecular dynamics simulation based on consensus from ligand-based and structure-based methods.

#### The docking protocol was validated with a known antagonist (UR-MK299) and PC compounds were observed to bind at the orthosteric binding site of Y_1_R with favourable binding energies

Docking protocol validations demonstrated that UR-MK299 bound favourably to Y_1_R at the canonical binding site (blind docking affinity: -11.2 kcal/mol, focused docking affinity: -11.7 kcal/mol). As observed in S3 Fig, the hydrogen bonds (with Asn283 and Asp287), pi-stacking contacts (with Phe282 and Phe286), and disulphide bridges (with Cys121) were reflected in both the re-docked experiments, consistent with previously reported PPARγ co-crystal structures (S3 Fig). Applying the validated protocols, 44 compounds, identified from TCM-ID and TCMSP databases, were docked with Y_1_R (PDB: 5ZBQ). A total of 396 docking poses, and binding energies were obtained, and an allosteric site within the transmembrane domain was identified through blind docking (Fig 1g–1j). Strikingly, all PC compounds in both blind and focused docking were scored with a negative (favourable) binding affinity, as shown in Table 1. Consistent with other docking studies [21] and the positive control UR-MK299, the median energy scores from focused docking (-10.1 kcal/mol) within the pre-defined box size were significantly more superior compared to blind docking (-7.95kcal/mol), and hence PC compounds in focused docking were selected for further analyses in the next few sections.

**Table 1:** Binding affinity results, calculated K*i* values, and residue contacts from focused docking (kcal/mol) of PC ligands with Y_1_R (PDB: 5ZBQ).

CID	Index	Compound name	Formula	ΔG _(kcal/mol)_	K_*i* (μM)_	Weight _(g/mol)_	Hydrogen bonds	Hydrophobic contacts
15391339	PC37	3beta-p-Hydroxybenzoyldehydrotumulosic acid	C_38_H_52_O_6_	-12.6	0.001	604.8	Asp104, Asp200, Asn283	Phe28, Asp104, Phe173, Val197, Phe199, Leu215, Phe282, Phe286, Ala294, His298
92156	PC12^#^	beta-Amyrin acetate	C_32_H_52_O_2_	-12.2	0.001	468.8	Gln219	Pro117, Gln120, Ile124, Phe173, Phe199, Trp276, Leu279, Phe302
9805290	PC25	Polyporenic acid C	C_31_H_46_O_4_	-11.9	0.002	482.7	Asn299	Leu26, Phe28, Thr97, Tyr100, Asp104, Pro117, Val197, Phe199, Phe282, Ala294, Phe302
15226717	PC35	Dehydropachymic acid	C_33_H_50_O_5_	-11.9	0.002	526.7	NA	Leu26, Phe28, Thr97, Tyr100, Asp104, Pro117, Gln120, Val197, Phe199, Phe282, Ala294, Phe302
444679	PC16	Ergosterol	C_28_H_44_O	-11.8	0.002	396.6	NA	Leu26, Phe28, Asp31, Asp104, Phe184, Val197, Phe199, Phe286, Ala294, Phe302
10743008	PC30	16alpha-Hydroxydehydrotrametenolic acid	C_30_H_46_O_4_	-11.6	0.003	470.7	Asn116, Thr295	Leu26, Phe28, Thr97, Tyr100, Pro117, Val197, Phe199, Phe282, Ala294, Phe302
15391340	PC38	3-Dehydrotrametenolic acid	C_30_H_46_O_3_	-11.6	0.003	454.7	Asn116, Asn299	Leu26, Phe28, Thr97, Tyr100, Pro117, Val197, Phe199, Phe282, Phe302
5283628	PC18	Stellasterol	C_28_H_46_O	-11.4	0.004	398.7	Asp104	Phe28, Val197, Phe199, Phe282, Asn283, Phe286, Ala294, Phe302
5484385	PC23	Pachymic acid	C_33_H_52_O_5_	-11.1	0.007	528.8	Asp104, Asn299	Leu26, Phe28, Tyr100, Asp104, Phe173, Phe184, Val197, Phe282, Asn283, Phe302
21159065	PC39	See below^a^	C_32_H_48_O_5_	-11.1	0.007	512.7	Asp104, Asn299	Phe28, Thr97, Tyr100, Asp104, Phe184, Val197, Phe199, Phe282, His298, Phe302
10005581	PC26^#^	3-Epidehydrotumulosic acid	C_31_H_48_O_4_	-11	0.008	484.7	Asp104, Asn299	Leu26, Phe28, Tyr100, Asp104, Pro117, Phe173, Phe184, Val197, Asn283, Phe302
12309443	PC32	Trametenolic acid	C_30_H_48_O_3_	-11	0.008	456.7	Asp287, Asn299	Leu26, Phe28, Asp104, Phe184, Val197, Phe199, Asn283, Phe286, Phe302
10368709	PC29	25-Hydroxy-3-epidehydrotumulosic acid	C_31_H_48_O_5_	-10.9	0.010	500.7	Asp287, Asn299	Leu26, Phe28, Asp104, Phe199, Asn283, Phe286, Phe302
12314446	PC33	Tumulosic acid	C_31_H_50_O_4_	-10.9	0.010	486.7	Asp104, Asn299	Leu26, Phe28, Asp104, Val197, Phe199, Phe282, Asn283, Phe286, Phe302
73402	PC10	Eburicoic acid	C_31_H_50_O_3_	-10.8	0.012	470.7	Asp104, Asn299	Leu26, Phe28, Asp104, Asn283, Phe286, Ala294, Phe302
10181133	PC27^#^	Cerevisterol	C_28_H_46_O_3_	-10.8	0.012	430.7	Pro117, Asn283	Phe28, Thr97, Tyr100, Thr101, Gln120, Val197, Phe199, Ala294, Phe302
5471852	PC21^#^	Poricoic acid B	C_30_H_44_O_5_	-10.7	0.014	484.7	Asp104, Gln177, Asp200, Asn299	Phe28, Asp104, Val197, Phe199, Phe282, Asn283, Phe302
15225964	PC34	Dehydrotumulosic acid	C_31_H_48_O_4_	-10.7	0.014	484.7	His298, Asn299	Leu26, Phe28, Asp104, Val197, Phe199, Phe282, Asn283, Phe286, Phe302
5471966	PC22^#^	Poricoic acid G	C_30_H_46_O_5_	-10.6	0.017	486.7	Asp104, Gln177, Asp200, Asn299	Leu26, Phe28, Asp104, Val197, Phe199, Phe282, Asn283, Phe286, Phe302
15250826	PC36	Dehydroeburicoic acid	C_31_H_48_O_3_	-10.6	0.017	468.7	Asn299	Leu26, Phe28, Asp104, Phe184, Val197, Phe199, Asn283, Phe286, Phe302
5471851	PC20	Poricoic acid A	C_31_H_46_O_5_	-10.5	0.020	498.7	Gln177, Asp200, His298, Asn299	Leu26, Phe28, Phe184, Val197, Phe199, Phe282, Asn283, Phe286, Ala294, Phe302
72202422	PC42	See below^b^	C_34_H_54_O_5_	-10.5	0.020	542.8	Asp287, Asn299	Leu26, Tyr100, Pro117, Ile124, Phe173, Phe199, Phe282, Asn283, Phe286, Ala294, His298, Phe302
102378062	PC43	16alpha,25-Dihydroxy-24-methylene-3,4-secolanosta-4(28),7,9(11)-triene-3,21-dioic acid	C_31_H_46_O_6_	-10.3	0.028	514.7	Asp104, Gln177, Asp200, Asn299	Phe28, Phe282, Asn283, Phe286, Ala294, Phe302
10918099	PC31	poricoic acid H	C_31_H_48_O_5_	-10.2	0.033	500.7	Gln177, Asp200	Leu26, Phe28, Phe282, Asn283, Phe286, Phe302
56668247	PC41	Poricoic acid C	C_31_H_46_O_4_	-10.1	0.039	482.7	Gln177, Asp200, Asn299	Leu26, Phe28, Asp104, Phe184, Val197, Phe199, Phe282, Asn283, Phe286, Phe302
14697	PC9	Methyl dehydroabietate	C_21_H_30_O_2_	-10	0.046	314.5	NA	Leu26, Phe28, Phe199, Phe282, Phe286, Ala294, Phe302
1203	PC5	(+/-)-Catechin	C_15_H_14_O_6_	-9	0.249	290.27	Asp104, His298, Asn299	Leu26, Phe28, Asp104, Val197, Phe199, Ala294
182232	PC15	(+)-Epicatechin	C_15_H_14_O_6_	-8.9	0.294	290.27	Asn283, Asn299	Leu26, Phe28, Phe199, Phe282, Phe286, Ala294, His298, Asn299
6441913	PC24	Coniferyl ferulate	C_20_H_20_O_6_	-8.9	0.294	356.4	Gln177, Cys198, Asp200	Leu26, Phe28, Asp104, Phe184, Val197, Phe199
5319022	PC19	Ligustilide	C_12_H_14_O_2_	-8	1.347	190.24	NA	Phe28, Phe199, Phe282, Phe286, Ala294, His298
40428662	PC40^#^	1-(alpha-L-Ribofuranosyl)uracil	C_9_H_12_N_2_O_6_	-6.6	14.348	244.2	Thr212, Gln219, Leu279, Asn283, Asp287	NA
121667	PC13	ethyl beta-D-glucopyranoside	C_8_H_16_O_6_	-6.4	20.117	208.21	Gln219, Thr280, Asn283	Phe173
72	PC1^#^	3,4-Dihydroxybenzoic acid	C_7_H_6_O_4_	-6.3	23.820	154.12	Thr212, Leu216, Asn283, Asp287	Asn283
985	PC4	Palmitic acid	C_16_H_32_O_2_	-6.3	23.820	256.42	Glu29, Asn30	Phe28, Tyr100, Val197, Phe199, Phe282, Phe286, Ala294
8468	PC8^#^	Vanillic acid	C_8_H_8_O_4_	-6.2	28.205	168.15	Thr212, Asn283, Asp287	Leu216, Tyr220, Thr280
5282729	PC17	2-Dodecenoic acid	C_12_H_22_O_2_	-6.2	28.205	198.3	Glu29, Asn30, Ala294	Phe28, Phe199, Phe282, Phe286
3893	PC6	Lauric acid	C_12_H_24_O_2_	-5.9	46.824	200.32	Asp104	Phe28, Val197, Phe282, Phe286, Ala294, His298, Phe302
74112	PC11	Trimethyl citrate	C_9_H_14_O_7_	-5.6	77.734	234.2	Asp104, Asn299	Phe28, Phe199, Ala294
8180	PC7	Undecanoic acid	C_11_H_22_O_2_	-5.4	108.988	186.29	Thr295	Leu26, Phe199, Phe282, Phe286, Ala294, His298, Phe302
125207	PC14	11-Dodecenoic acid	C_12_H_22_O_2_	-5.4	108.988	198.3	Asn299	Leu26, Phe199, Phe282, Phe286, Ala294, His298, Phe302
190	PC2	Adenine	C_5_H_5_N_5_	-5.3	129.050	135.13	Gln219, Leu279, Thr280, Asn283	NA
379	PC3	Octanoic acid	C_8_H_16_O_2_	-5.1	180.935	144.21	Glu29, Asn30, Ala294	Phe28, Asp104, Val197, Phe199, Ala294
10285815	PC28	(S)-Dimethyl 2-hydroxysuccinate	C_6_H_10_O_5_	-5.1	180.935	162.14	Gln219, Asp287	Thr280

NA indicates no hydrogen bonds or hydrophobic residues. Each PC index represents a *Poria cocos* compound. Marked IDs^#^ are PC compounds with putative antagonist roles, as discussed in text. Binding affinity scores were generated by AutoDock Vina (v1.1.2). Inhibition constant (Ki) = exp(ΔG×1000/RT), where ΔG is the docking energy, R is 1.985 x 10^−3^ kcal/mol/K and T is 298K.

^a^(2R)-2-[(3S,5R,10S,13R,14R,16R,17R)-16-acetyloxy-3-hydroxy-4,4,10,13,14-pentamethyl-2,3,5,6,12,15,16,17-octahydro-1H-cyclopenta[a]phenanthren-17-yl]-6-methylhept-5-enoic acid.

^b^methyl (2R)-2-[(3S,5R,10S,13R,14R,16R,17R)-3-acetyloxy-16-hydroxy-4,4,10,13,14-pentamethyl-2,3,5,6,7,11,12,15,16,17-decahydro-1H-cyclopenta[a]phenanthren-17-yl]-6-methyl-5-methylideneheptanoate. Distances of hydrogen and hydrophobic contacts are listed in S2 and S3 Tables, respectively.

### Hydrogen bonds between PC compounds and key residues of Y_1_R binding site within the orthosteric ligand-binding domain and the deep V-shaped sub-pocket

**Fig 2:**
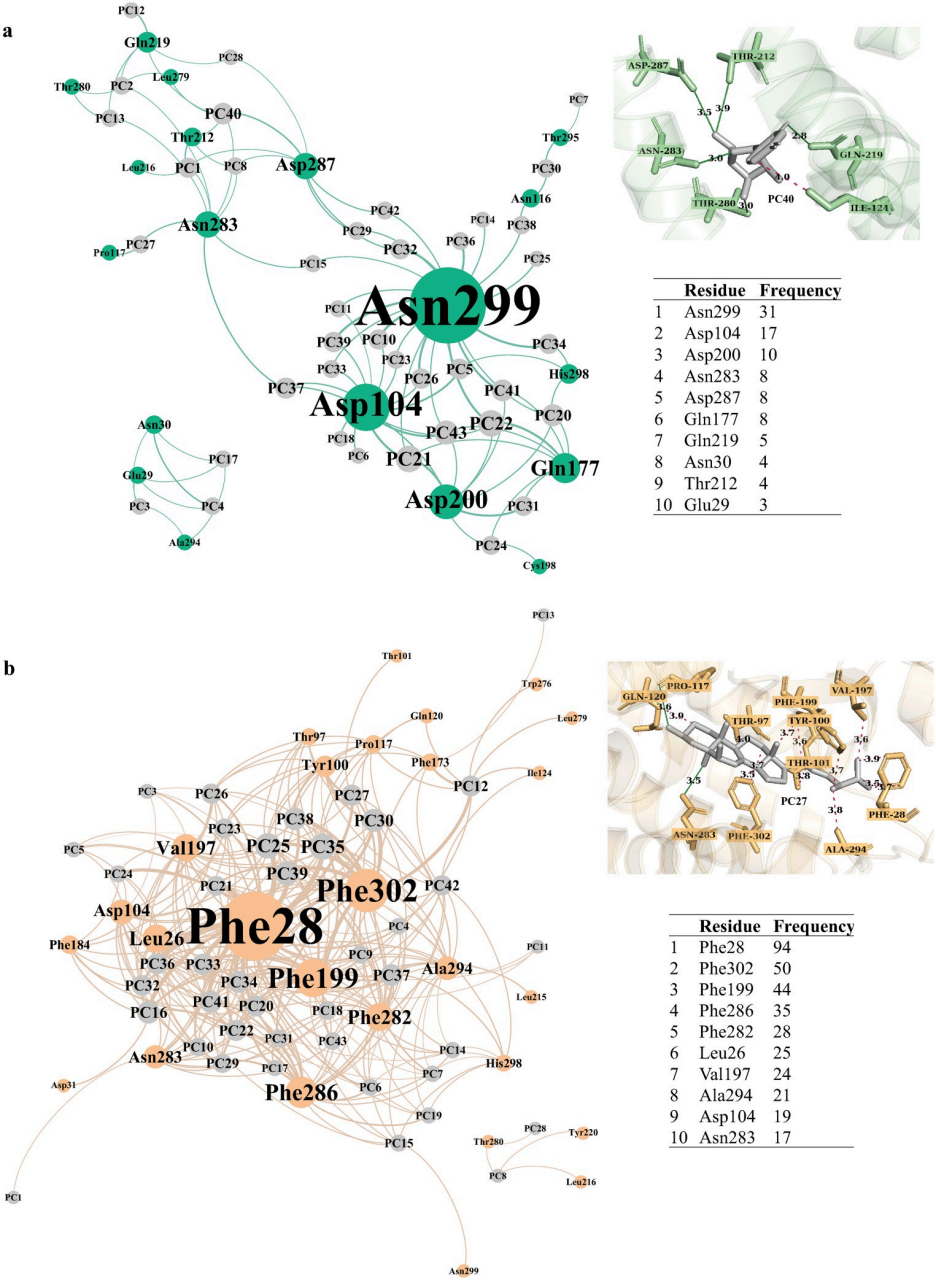
3D ligand-residue interactions outlining hydrogen bonds (a) and hydrophobic interactions (b) in focused docking of *Poria cocos* compounds and Y_1_R. The node sizes of PC compounds (grey) and Y_1_R residues are based on the number of degrees, i.e., the higher the frequency of connections between residues and PC compounds, the larger the node size.

A high frequency (≥ 5) of hydrogen bonds between PC compounds and key polar or charged residues were observed (Fig 2a), including Asn299 (*n* = 31), Asp104 (*n* = 17), and Asp200 (*n* = 10), located near the extracellular domain, and Asn283 (*n* = 8), Asp287 (*n* = 8) found deep in the Y_1_R sub-pocket. Distance information on hydrogen bond interactions is provided in S2 Table.

#### Orthosteric ligand-binding domain

As a residue with a high propensity to donate and accept hydrogen due to its carboxylate and amide side chains, it is not surprising to observe a large majority (48.8%) of PC compounds form polar contacts with Asn299. Although the role of Asn299 in Y_1_R is not yet well known, studies on Asn located in a similar location (near extracellular loop of helix VII) of other class A GPCRs, the M2 muscarinic acetylcholine receptor, suggest that a mutation from Asn to Cys (as well as Val to Cys) significantly reduced binding affinity of the orthosteric antagonist [51]. Similarly, Asp104 located in helix II could also play important roles in stabilising extracellular loop (ECL) 2 and affect ligand-binding activities, as the substitution of Asp104 to Ala completely abolished the binding of endogenous peptide, NPY [52]. In addition, the importance of Asp200 in endogenous ligand binding has been demonstrated in docking studies between peptide YY (PYY) and Y_1_R, as a hydrogen bond was indicated with 1.8Å between the peptide and receptor residue Asp200 [53]. A study on Asp200 conducted by Walker et al. (1994) [52] also revealed that a mutation of Asp200 to Ala completely abolished ^125^1-NPY binding activities at 50pM concentration. However, these findings contrasted with a later study conducted by Sautel et al. (1996) [54], which they found Asp200 to Ala mutation exhibited similar K_*i*_ values as the wild-type receptor for either the endogenous ligand (NPY) or non-peptide antagonist (BIBP 3226). Asp200 may therefore serve an indirect role to support binding events, pending confirmation with more recent investigations. Nevertheless, PC compounds (**PC21**, **PC22** and **PC43**) interacting with Asn299, Asp104 and Asp200 by hydrogen bonds, could interfere with agonist binding and stabilising ECL2 in a closed position.

#### Deep V-shaped sub-pocket

Within the deep sub-pocket, two highly important residues for maintaining inactive conformation of Y_1_R are Asn283 on helix V and Asp287 on helix VI. These residues have been well studied for their ligand-recognition effects on Y_1_Rs. PC compounds forming hydrogen bonds with both Asn283 and Asp287 were **PC1**, **PC8** and **PC40**. Mutagenesis studies indicate that alanine mutations of Asn283 and Asp287 both showed no or significantly lower binding of both endogenous agonist (NPY) and non-peptide antagonist (BIBP3226) [52, 54]. This observation is consistent with recent structural studies [25], by which the binding affinity scores of the antagonist UR-MK299 (positive control in this study) were reduced by over 2,000-fold when Asn283 and Asp287 were mutated to Ala and Asp residues, respectively. Regardless of peptide or non-peptide ligands, these studies strongly support the roles of Asn283 and Asp287 in substrate binding. The individual and synergistic roles of these residues on receptor function and the selectivity of peptide and non-peptide molecules on Y_1_R could be further explored.

### Extensive contacts with conserved and non-conserved Phe residues within the hydrophobic cluster and may interfere with binding of endogenous ligands near the extracellular surface of Y_1_R

Aside from hydrogen bonding residues, PC compounds were stabilised by hydrophobic contacts with Y_1_R residues (Fig 2b), their distances were provided in S3 Table. The most targeted hydrophobic contacts within 4Å were Phe residues on extracellular loops (Phe28 and Phe199) and on transmembrane helices V and VI (Phe282, Phe286 and Phe302). As we aimed to identify possible inhibitors of Y_1_R, only PC compounds contacting the hydrophobic cluster indicated for Y_1_R antagonism were discussed. Among these hydrophobic clusters, it has been revealed that alanine mutation of the non-conserved Phe286 resulted in significantly lowered binding affinity for both agonist (NPY) and antagonist (BIBP3266) [54]. More specifically, Phe286 mutations to Ala resulted in a 3-fold increase in K_*d*_ values. Furthermore, Phe282 and Phe286 have also been implicated for G protein coupling, as substitutions of these residues aborted the activity of PYY without significant changes to the binding affinity of antagonists 1229U91 and J-104870 [55]. In contrast, alanine mutation of Phe302 has resulted in the complete abolishment of activities from known antagonists BMS-193885, BIBP3226, BIBO3304, UR-MK136 and UR-MK289 [25]. These findings suggest that Phe302 makes a profound impact on antagonistic activities of Y_1_R, and those compounds that solely interact with Phe302 were **PC12**, **PC26** and **PC27** (S4 Fig).

### A consensus approach identified PC12, PC26 and PC27 as putative Y_1_R inhibitors

Following ligand- and structure-based screening, close examination of protein-ligand contacts at the important binding site residues identified several PC compounds for further analysis, as listed in Table 2. Considering the binding energies, drug-likeness profiles, and toxicity indexes, PC12, PC26 and PC27 could be proposed as candidate compounds targeting Y_1_R and is worth further investigation. PC12, beta-amyrin acetate was selected as the compound for molecular dynamics simulations as it produced the highest binding score out of the three.

**Table 2:** Putative Y_1_R inhibitors identified from ligand-based and structure-based screening approaches.

Putative Y_1_R inhibitors	ΔG (kcal/mol)	Drug-likeness score	AMES toxicity (yes/no)	Mutagenic (high/none/low)	Tumour-genic (high/none/low)	Irritant (high/none/low)
PC1	-6.3	-1.8	No	high	none	none
PC8	-6.2	-1.6	No	high	none	none
PC12*	-12.2	-2.7	No	none	none	none
PC21	-10.7	-21.0	No	none	none	none
PC22	-10.6	-21.3	No	none	none	none
PC26*	-11.0	-6.5	No	none	none	none
PC27*	-10.8	-0.3	No	none	none	none
PC40	-6.6	-4.6	No	none	none	none
PC43	-10.3	-14.6	No	none	none	high

*Candidate compounds as putative Y_1_R inhibitors.

### Structural analyses from 100 ns of MDS trajectories suggest reasonable stability of PC12-Y_1_R complex

**Fig 3:**
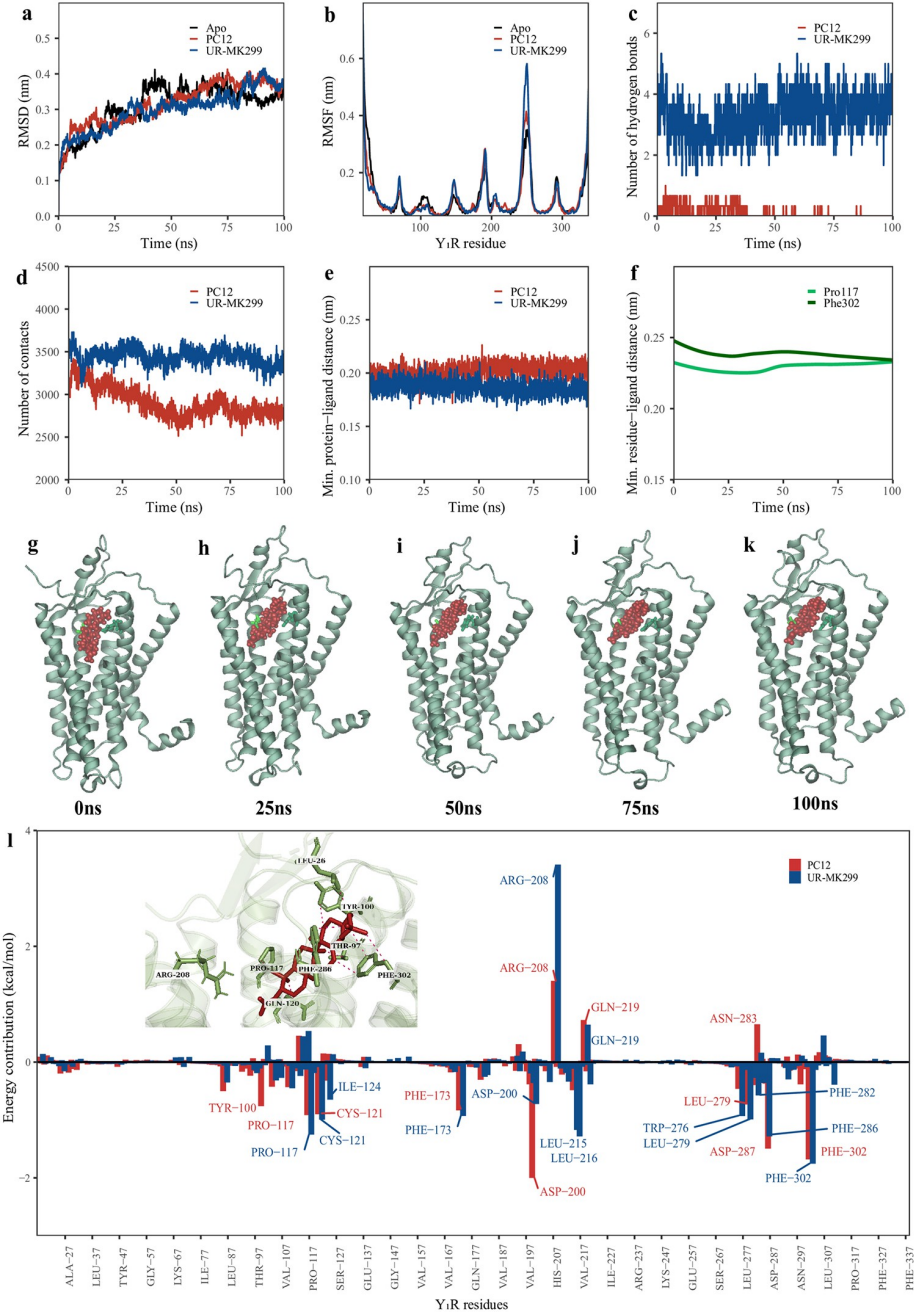
Structural analyses, snapshots of trajectories and binding free energy calculations from molecular dynamics simulation comparing PC12 (red) with its positive control UR-MK299 (blue) on Y_1_R (green). Structural analyses include RMSD (a), RMSF (b), number of hydrogen bonds (c), number of contacts (d), minimum protein-ligand distances (e), and minimum distance between residues (Pro117, Phe302) on either side of the ligand (PC12) (f). Snapshots of Y_1_R (pale green cartoon) and PC12 (red CPK) visualised in VMD throughout 100 ns (g-k). Per-residue energy decomposition for PC12 and UR-MK-299 (l), with a 3D ligand-residue inset of PC12 (red line and sticks).

The conformational dynamics on stability and fluctuations were examined with RMSD, RMSF, number of hydrogen bonds, number of contacts, minimum protein-ligand distance, and minimum residue-ligand distance. In all simulations, the RMSD increased steadily and reach a plateau by approximately 35ns. After reaching equilibrium, the average root mean-squared deviations (RMSD) of backbone atoms fitted to the starting structure (t = 0) for the apo-, PC12- and UR-MK299-Y_1_R complexes were 0.331nm, 0.339nm and 0.319nm, respectively (Fig 3a). Interestingly, PC12-Y_1_R appears to have marginally higher RMSD compared to UR-MK299- Y_1_R, this is attributed to the drift in the intracellular loop 3 of the second and third MD runs. As expected, most movements occurred in the extracellular and intracellular loop regions, while the transmembrane helices were relatively stable. Furthermore, the magnitude of RMSD were comparable to other rhodopsin-like GPCRs suspended in a POPC membrane system, with an average of approximately 0.35nm [56]. Per-residue fluctuation across the trajectory (Fig 3b) revealed several major peaks which corresponds to the loop regions, consistent with the RMSD trends. The highest peaks with the greatest flexibility among all simulations were between Lys239 and Thr258, the longest intracellular loop connecting helices 5 and 6. No significant differences between the RMSF peaks of Y_1_R were apparent, although a marginally lower average RMSF could be seen with the PC12-bound Y_1_R (red) compared to the UR-MK299-bound Y_1_R (blue), suggestive of a stronger loop-stabilising effect of PC12.

For both UR-MK299 and PC12 complexes after equilibrium, the number of hydrogen bonds remained relatively constant throughout the trajectory (Fig 3c), despite a slight decline in numbers for PC12 shortly after system relaxation. As the number of hydrogen bonds for PC12 were comparatively low, further assessments on number of contacts and protein-ligand distances were performed to examine whether protein-ligand interactions were present throughout the 100 ns trajectory. Intriguingly, the differences in number of contacts (Fig 3d) and the minimum protein-ligand distances (Fig 3e) for PC12 (2988 and 0.202nm, respectively), appear comparable to that of UR-MK299 (3443 and 0.187nm, respectively). Similarly, the distances between Pro117 (on helix 3) and Phe302 (on helix 7), two residues situated at either side of PC12 in Fig 3f appeared consistent at approximately 0.23 to 0.24nm across the smoothed trajectory. As such, the proximity of PC12 with Y_1_R residues indicates that the ligand remained bound and fitted well within the orthosteric ligand-binding domain. The stable and sustained interactions may be governed by non-polar contacts such as pi-stacking interactions, in stark contrast with the binding patterns of the positive control UR-MK299. Snapshots of the trajectories in Fig 3g–3k shows PC12-bound Y_1_R at different time series (ns), aside from a small shift in ligand orientation, PC12 remains bound to the orthosteric binding domain.

### Binding free energy estimated by MMPBSA indicate that PC12 could bind with relatively strong free energies to Y_1_R

The stability of **PC12** and its positive control (UR-MK299) was further assessed by the energy contributions from electrostatic, van der Waals, polar solvation and SASA terms using MMPBSA. Table 3 lists the components of binding energies (kcal/mol) averaged over three parallel simulations for binding of **PC12** and UR-MK299 to the inactive Y_1_R. Interestingly, **PC12** revealed stronger binding energies (ΔG_binding_ = -36.1 kcal/mol) compared to the positive control UR-MK299 (ΔG_binding_ = -32.4 kcal/mol), where most interactions were driven by van der Waals forces in both systems. The major difference in binding energies was attributed to the lower positive polar solvation energy arising from binding with PC12 (14.8 kcal/mol), compared to UR-MK299 (56.3 kcal/mol). Consistently, using the same binding free energy calculation approach, the ΔG_binding_ of PC12 beta-amyrin acetate appears in agreement to its pentacyclic triterpenol variant, alpha-amyrin acetate, bound to cannabinoid type 1 receptor (-36.6 kcal/mol) [57]. Whether PC12 or UR-MK299 were able to compete with the endogenous ligands NPY or PYY to inactive Y_1_R remains to be researched, as the ΔG_binding_ of PYY was found to be -50.6 kcal/mol, a significantly more favourable affinity compared to small molecules [53]. Nevertheless, the results suggest that PC12 could bind to the orthosteric ligand binding domain of Y_1_R with relatively strong binding free energies.

**Table 3:** Components of binding energies (kcal/mol) averaged over three parallel simulations.

Components of binding energies (kcal/mol)	PC12	UR-MK299
ΔG_vdw_	-45.1	-65.5
ΔG_elec_	-0.3	-15.7
ΔG_psolv_	14.8	56.3
ΔG_SASA_	-5.5	-7.6
ΔG_binding_	-36.1	-32.4

vdw, van der Waals interactions; elec, electrostatic energy; psolv, polar solvation energy; SASA, solvent-accessible surface area.

To examine the relative importance of residues for ligand binding at the orthosteric site, the total energy contribution of each system was decomposed into each corresponding residue. The average per-residue binding free energies for the final 1ns of three trajectories is shown in Fig 3l. Trends in per-residue binding energies were mostly consistent between **PC12** and UR-MK299. The most energetically favourable residues tended to be non-polar and aggregate around helix III (Pro117, Cys121), helix VI (Phe173), helix VI (Phe282, Phe286) and helix VII (Phe302). Interestingly, two charged asparagine residues on ECL2 and helix IV were involved in significantly stronger binding free energies for **PC12** (Asp200: -1.98 kcal/mol, Asp287: -1.48 kcal/mol) compared to UR-MK299 (Asp200: -0.71 kcal/mol, Asp287: 0.05 kcal/mol). The contrasting contributions from Asp residues may be important for the selectivity of **PC12** or PC compounds on Y_1_R. Notably, the hydrophobic cluster governed by Phe282, Phe286 and Phe302 is also apparent in previous experimental studies on known Y_1_R antagonists UR-MK299 and BMS-193885 [25, 58]. The most favourable contribution from Phe302 among the cluster (**PC12**: -1.67 kcal/mol, UR-MK299: -1.74 kcal/mol) is consistent with findings from the protein-ligand interaction analyses earlier, and warrants mutagenesis studies to confirm its role on receptor inactivation. As PC12 demonstrated strong hydrophobic interactions with residues in the hydrophobic cluster, this could serve as a pathway for stabilising the Y_1_R conformation in the inactive state.

In contrast to the residues above, Arg208 and Gln219 were found to contribute to positive energies at the orthosteric binding site. The binding free energies of the basic residue, Arg208, appeared to be highly positive for UR-MK299 (3.40 kcal/mol) and to a slightly lesser extent for PC12 (1.39 kcal/mol). The presence of the basic residue may contribute to conformational changes at the apical surface of Y_1_R, which could be supported by charged-assisted hydrogen bonds when interacting with endogenous peptides or small molecules. This mechanism has also been discussed in an early study by Sylte et al. [59], demonstrating the role of Arg208 on the stability of ECL2 through intramolecular electrostatic interactions with Asp182 and Asp205. In addition, the binding free energies of Gln219 were also positive when bound to UR-MK299 (0.63 kcal/mol) and PC12 (0.71 kcal/mol). Consistent with UR-MK299 and PC12 in our study, polar interactions with Gln219 were not found to interact with antagonist BIBP3226, but was found to be within the binding region with Tyr36 of NPY, suggesting that this residue could be important for receptor activation [60]. Although Arg208 (helix V) and Gln219 (ECL 2), were not found to be within close contact with the ligands at the stabilised conformations throughout the 100 ns simulation, these conserved residues among neuropeptide Y receptor subtypes (Y_4_R, Y_5_R) could be important for ligand recognition as they were located the extracellular surface, pending further confirmation with future mutagenesis studies.

## Conclusion

In summary, this study was conducted to critically examined the pharmacokinetics profiles of PC compounds and to explore their molecular mechanisms targeting Y_1_R. We discovered several putative Y_1_R inhibitors using ligand- and structure-based approaches, which could be used as starting points for further development, optimisation, and refinement as anti-obesity agents. Compounds designated **PC1** 3,4-Dihydroxybenzoic acid, **PC8** Vanillic acid, **PC40** 1-(alpha-L-Ribofuranosyl)uracil were proposed as potential antagonists as they contact major residues Asn283 and Asp287, similar to various potent Y_1_R antagonists. **PC21** Poricoic acid B, **PC22** Poricoic acid G and **PC43** 16alpha,25-Dihydroxy-24-methylene-3,4-secolanosta-4(28),7,9(11)-triene-3,21-dioic acid, contacting Asn299, Asp104 and Asp200 proximal to the extracellular surface could also interfere with agonist binding by stabilising the extracellular loop (ECL) 2 of Y_1_R in a closed position. Owing to their selective interaction with Phe302, an important residue in binding of selective Y_1_R antagonists, **PC12** beta-Amyrin acetate, **PC26** 3-Epidehydrotumulosic acid and **PC27** Cerevisterol were also suggested to be putative antagonists. Following the consensus approach from ligand- and structure-based screening, candidate compounds **PC12**, **PC26** and **PC27** were identified due to their high affinities (-12.2, -11.0 and -10.8 kcal/mol, respectively), high drug-likeness and low toxicity profiles. In combination with molecular dynamics simulations and binding free energy calculations, we achieved successful validation of the binding feasibility of **PC12**, as shown by its structural stability and favourable binding free energies at the orthosteric Y_1_R transmembrane domain.

As relatively few studies adopted multiple virtual screening strategies, the consensus framework from ligand- and structure-based approaches with subsequent validation of feasibility in molecular dynamics simulations makes this combinatorial approach a strength of this study. Aside from binding affinity values, binding poses and interactions, the pharmacokinetics properties were also examined, offering room for lead optimisation and refinement to assist with a more successful design of PC compounds as more potent Y_1_R inhibitors. Furthermore, the application of high-throughput screening and analyses methods not only enables more efficient identification of candidate PC compounds, but it also provides a comprehensive understanding of ligand-residue interactions, binding characteristics and pharmacological regulation globally, at the herb level, in addition to many studies solely reporting actions of selected compounds from *Poria cocos*. Although findings from molecular dynamics simulations corroborate the accuracy of results from docking analyses, they were only performed for a relatively short duration and in triplicate. More computationally intensive simulations up to and beyond microsecond timescales with higher number of parallel runs may reveal additional behaviours of ligand-protein complexes, including ligand-entry and possible concerted motions between other functionally relevant regions and the allosteric sites, not sampled by the 100 ns simulations in this study. Finally, as a limitation among computational studies, the predictive binding affinities, inhibitory constants, and pharmacokinetic properties may not be reflective of experimental values. Therefore, further investigations from *in vitro* and *in vivo* studies are desired to validate, characterise, and expand on the actions induced by PC compounds on Y_1_R for future obesity management.

## Supporting information

S1 Table Protein-ligand interaction profiler thresholds for each parameter. The mode of analysis was specified as “detection of macromolecule-ligand interactions”.
(PDF)

S2 Table Distance of hydrogen contacts between PC compounds and Y_1_R residues. H-A, Distance between H-Bond hydrogen and acceptor atom (Å); D-A, Distance between H-Bond donor and acceptor atoms (Å).
(PDF)

S3 Table Distance of hydrophobic contacts between PC compounds and Y_1_R residues. Distance provided in Angstrom (Å).
(PDF)

S1 Fig 2D conformer structures of all 43 PC compounds. Within each compound box, the following information were provided: PC index (top left), chemical formula (top right), PubChem CID (bottom center).
(PDF)

S2 Fig Overview of ADMET properties of PC compounds based on pkCSM outputs. For each continuous parameter, molecular weight was plotted in the x-axis against the parameter. Red line shows suggested thresholds for continuous parameters, where applicable.
(PDF)

S3 Fig Best binding poses of three molecules of co-crystalised known antagonist UR-MK299 on Y_1_R (PDB:5ZBQ). Ligand residue interactions in 3D and 2D views. (a) 3D diagram of 9AO superimposed in Y_1_R (green ribbons). (b) 2D diagram of 9AO (grey sticks) in its original co-crystallised form. (c) 2D diagram of 9AO (orange sticks) in blind docking. (d) 2D diagram of 9AO (yellow sticks) in focused docking.
(PDF)

S4 Fig Interaction distance between *Poria cocos* compounds and residues of the hydrophobic phenylalanine (Phe) cluster in focused docking. The colour of tiles represents an interaction detected within 4Å (green), and more than 4Å (white). The colour gradient represents the distance between the Phe residues and *Poria cocos* compounds, the closer the distance the darker the colour.
(PDF)
