# Supplementary material for: Poria cocos compounds targeting neuropeptide Y1 receptor (Y1R) for weight management: A computational ligand- and structure-based study with molecular dynamics simulations identified beta-amyrin acetate as a putative Y1R inhibitor
Source: PLoS One. 2023 Jun 30;18(6):e0277873. doi: 10.1371/journal.pone.0277873 (PMC10313034; doi:10.1371/journal.pone.0277873)
Supplement: S1 Table — The mode of analysis was specified as “detection of macromolecule-ligand interactions”. (PDF) [file pone.0277873.s001.pdf]

S1 Table. Protein-ligand interaction profiler thresholds for each parameter.

Parameters	Threshold	Units
aromatic_planarity	5.0	°
hydroph_dist_max	4.0	Å
hbond_dist_max	4.1	Å
hbond_don_angle_min	100.0	°
pistack_dist_max	5.5	Å
pistack_ang_dev	30.0	°
pistack_offset_max	2.0	Å
pication_dist_max	6.0	Å
halogen_dist_max	4.0	Å
halogen_acc_angle	120.0	°
halogen_don_angle	165.0	°
halogen_angle_dev	30.0	°
saltbridge_dist_max	5.5	Å
water_bridge_mindist	2.5	Å
water_bridge_maxdist	4.1	Å
water_bridge_omega_min	71.0	°
water_bridge_omega_max	140.0	°
water_bridge_theta_min	100.0	°

The mode of analysis was specified as “detection of macromolecule-ligand interactions”.
