# Supplementary material for: Poria cocos compounds targeting neuropeptide Y1 receptor (Y1R) for weight management: A computational ligand- and structure-based study with molecular dynamics simulations identified beta-amyrin acetate as a putative Y1R inhibitor
Source: PLoS One. 2023 Jun 30;18(6):e0277873. doi: 10.1371/journal.pone.0277873 (PMC10313034; doi:10.1371/journal.pone.0277873)
Supplement: S2 Table — H-A, Distance between H-Bond hydrogen and acceptor atom (Å); D-A, Distance between H-Bond donor and acceptor atoms (Å). (PDF) [file pone.0277873.s002.pdf]

S2 Table. Distance of hydrogen contacts between *Poria cocos* compounds and Y_1R residues.

Index	Residue	Distance H-A	Distance D-A	Type of interaction
PC1	Thr212	2.85	3.21	hydrogen
PC1	Thr212	2.28	3.21	hydrogen
PC1	Leu216	2.98	3.34	hydrogen
PC1	Asn283	2.38	2.95	hydrogen
PC1	Asp287	2.38	2.89	hydrogen
PC2	Gln219	2.81	3.82	hydrogen
PC2	Leu279	3.42	4.1	hydrogen
PC2	Thr280	2.47	3.15	hydrogen
PC2	Asn283	1.94	2.93	hydrogen
PC3	Glu29	2.77	3.27	hydrogen
PC3	Asn30	2.16	2.86	hydrogen
PC3	Ala294	1.94	2.83	hydrogen
PC4	Glu29	3.28	3.91	hydrogen
PC4	Asn30	2.62	3.16	hydrogen
PC4	Asn30	2.14	2.96	hydrogen
PC5	Asp104	2.43	2.92	hydrogen
PC5	Asp104	2.11	2.92	hydrogen
PC5	His298	3.07	3.61	hydrogen
PC5	Asn299	3.29	4.04	hydrogen
PC6	Asp104	2.11	3.02	hydrogen
PC6	Asp104	2.38	3.02	hydrogen
PC7	Thr295	2.27	2.84	hydrogen
PC8	Thr212	2.33	2.71	hydrogen
PC8	Asn283	3.01	3.78	hydrogen
PC8	Asp287	3.03	3.57	hydrogen
PC10	Asp104	3.33	4.06	hydrogen
PC10	Asn299	2.42	3.22	hydrogen
PC10	Asn299	2.15	2.84	hydrogen
PC11	Asp104	1.8	2.72	hydrogen
PC11	Asn299	3.57	3.89	hydrogen
PC11	Asn299	2.72	3.15	hydrogen
PC12	Gln219	2.47	3.15	hydrogen
PC13	Gln219	3.01	3.97	hydrogen
PC13	Thr280	2.23	2.83	hydrogen
PC13	Asn283	2.43	3.39	hydrogen
PC14	Asn299	2.71	3.15	hydrogen
PC14	Asn299	2.77	3.1	hydrogen
PC15	Asn283	2.05	2.83	hydrogen
PC15	Asn299	1.99	2.75	hydrogen
PC17	Glu29	2.82	3.32	hydrogen
PC17	Asn30	2.17	2.87	hydrogen
PC17	Ala294	1.93	2.85	hydrogen
PC18	Asp104	3.2	3.72	hydrogen
PC20	Gln177	1.99	2.92	hydrogen
PC20	Asp200	2.18	3	hydrogen

Index	Residue	Distance H-A	Distance D-A	Type of interaction
PC20	His298	3.29	4.06	hydrogen
PC20	Asn299	2.36	3.19	hydrogen
PC21	Asp104	3.21	3.95	hydrogen
PC21	Asp104	2.1	3.01	hydrogen
PC21	Gln177	2.02	2.95	hydrogen
PC21	Asp200	2.36	3.15	hydrogen
PC21	Asp200	2.58	2.92	hydrogen
PC21	Asn299	2.49	3.27	hydrogen
PC22	Asp104	2.27	2.79	hydrogen
PC22	Gln177	2.04	2.96	hydrogen
PC22	Gln177	2.39	2.98	hydrogen
PC22	Asp200	2.38	3.16	hydrogen
PC22	Asn299	2.08	2.69	hydrogen
PC22	Asn299	2.45	3.24	hydrogen
PC23	Asp104	2.48	3.03	hydrogen
PC23	Asn299	2.44	3.26	hydrogen
PC24	Gln177	2.09	3.02	hydrogen
PC24	Cys198	3.29	3.78	hydrogen
PC24	Asp200	2.11	2.97	hydrogen
PC25	Asn299	2.73	3.21	hydrogen
PC26	Asp104	2.62	3.05	hydrogen
PC26	Asn299	2.59	3.15	hydrogen
PC26	Asn299	2.44	3.27	hydrogen
PC27	Pro117	3.01	3.56	hydrogen
PC27	Asn283	2.66	3.53	hydrogen
PC28	Gln219	2.2	2.95	hydrogen
PC28	Asp287	3.29	3.8	hydrogen
PC29	Asp287	2.56	3.49	hydrogen
PC29	Asn299	2.49	3.22	hydrogen
PC30	Asn116	2.13	2.99	hydrogen
PC30	Thr295	2.6	3.11	hydrogen
PC31	Gln177	2.11	3	hydrogen
PC31	Asp200	2.41	3.18	hydrogen
PC31	Asp200	2.5	2.89	hydrogen
PC32	Asp287	3.11	4.04	hydrogen
PC32	Asn299	2.32	3.14	hydrogen
PC32	Asn299	2.16	2.85	hydrogen
PC33	Asp104	2.33	2.72	hydrogen
PC33	Asn299	2.34	3.15	hydrogen
PC34	His298	3.63	4.04	hydrogen
PC34	Asn299	2.38	3.18	hydrogen
PC34	Asn299	1.94	2.8	hydrogen
PC36	Asn299	2.33	3.17	hydrogen
PC36	Asn299	2.03	2.9	hydrogen
PC37	Asp104	2.26	3.1	hydrogen
PC37	Asp200	3	3.83	hydrogen
PC37	Asn283	2.47	2.88	hydrogen

Index	Residue	Distance H-A	Distance D-A	Type of interaction
PC38	Asn116	2.55	3.06	hydrogen
PC38	Asn299	2.42	3.17	hydrogen
PC39	Asp104	2.65	3.45	hydrogen
PC39	Asn299	2.53	3.27	hydrogen
PC39	Asn299	2.17	2.69	hydrogen
PC40	Thr212	3.67	3.96	hydrogen
PC40	Gln219	2.16	3.07	hydrogen
PC40	Leu279	3.09	4.02	hydrogen
PC40	Asn283	2.03	3.03	hydrogen
PC40	Asp287	3.12	3.66	hydrogen
PC40	Asp287	2.96	3.66	hydrogen
PC41	Gln177	2.05	2.97	hydrogen
PC41	Asp200	2.33	3.13	hydrogen
PC41	Asn299	2.41	3.23	hydrogen
PC41	Asn299	2.34	2.93	hydrogen
PC42	Asp287	2.2	2.93	hydrogen
PC42	Asn299	3.33	3.87	hydrogen
PC43	Asp104	3.52	4.06	hydrogen
PC43	Asp104	2.22	2.93	hydrogen
PC43	Gln177	2.13	3.03	hydrogen
PC43	Asp200	2.35	3.14	hydrogen
PC43	Asn299	1.77	2.7	hydrogen

H-A, Distance between H-Bond hydrogen and acceptor atom (Å); D-A, Distance between H-Bond donor and acceptor atoms (Å).
