# Supplementary material for: Poria cocos compounds targeting neuropeptide Y1 receptor (Y1R) for weight management: A computational ligand- and structure-based study with molecular dynamics simulations identified beta-amyrin acetate as a putative Y1R inhibitor
Source: PLoS One. 2023 Jun 30;18(6):e0277873. doi: 10.1371/journal.pone.0277873 (PMC10313034; doi:10.1371/journal.pone.0277873)
Supplement: S3 Table — Distance provided in Angstrom (Å). (PDF) [file pone.0277873.s003.pdf]

S3 Table. Distance of hydrophobic contacts between *Poria Cocos* compounds and Y_1R residues.

**Index**	**Residues**	**Distance (Å)**	**Type of interaction**
PC1	Asn283	3.36	hydrophobic
PC3	Phe28	3.57	hydrophobic
PC3	Phe28	3.58	hydrophobic
PC3	Phe28	3.7	hydrophobic
PC3	Asp104	3.77	hydrophobic
PC3	Val197	3.69	hydrophobic
PC3	Phe199	3.87	hydrophobic
PC3	Ala294	3.71	hydrophobic
PC4	Phe28	3.52	hydrophobic
PC4	Phe28	3.79	hydrophobic
PC4	Phe28	3.86	hydrophobic
PC4	Tyr100	3.75	hydrophobic
PC4	Val197	3.61	hydrophobic
PC4	Phe199	3.6	hydrophobic
PC4	Phe282	3.74	hydrophobic
PC4	Phe286	3.71	hydrophobic
PC4	Phe286	3.79	hydrophobic
PC4	Ala294	3.65	hydrophobic
PC5	Leu26	3.53	hydrophobic
PC5	Phe28	3.98	hydrophobic
PC5	Asp104	3.76	hydrophobic
PC5	Val197	3.71	hydrophobic
PC5	Phe199	3.65	hydrophobic
PC5	Ala294	3.71	hydrophobic
PC6	Phe28	3.65	hydrophobic
PC6	Phe28	3.83	hydrophobic
PC6	Phe28	3.69	hydrophobic
PC6	Val197	3.55	hydrophobic
PC6	Phe282	3.57	hydrophobic
PC6	Phe282	3.59	hydrophobic
PC6	Phe286	3.65	hydrophobic
PC6	Phe286	3.65	hydrophobic
PC6	Phe286	3.76	hydrophobic
PC6	Ala294	3.7	hydrophobic
PC6	His298	3.69	hydrophobic
PC6	Phe302	3.98	hydrophobic
PC7	Leu26	3.94	hydrophobic
PC7	Phe199	3.8	hydrophobic
PC7	Phe282	3.92	hydrophobic
PC7	Phe282	3.63	hydrophobic
PC7	Phe286	3.73	hydrophobic
PC7	Phe286	3.98	hydrophobic
PC7	Ala294	3.63	hydrophobic
PC7	His298	3.74	hydrophobic
PC7	Phe302	3.78	hydrophobic

Index	Residues	Distance (Å)	Type of interaction
PC7	Phe302	3.7	hydrophobic
PC7	Phe302	3.64	hydrophobic
PC8	Leu216	3.63	hydrophobic
PC8	Tyr220	3.64	hydrophobic
PC8	Thr280	3.81	hydrophobic
PC9	Leu26	3.75	hydrophobic
PC9	Phe28	3.85	hydrophobic
PC9	Phe28	3.59	hydrophobic
PC9	Phe199	3.71	hydrophobic
PC9	Phe282	3.56	hydrophobic
PC9	Phe286	3.65	hydrophobic
PC9	Ala294	3.34	hydrophobic
PC9	Phe302	3.73	hydrophobic
PC9	Phe302	3.6	hydrophobic
PC10	Leu26	3.51	hydrophobic
PC10	Phe28	3.49	hydrophobic
PC10	Phe28	3.58	hydrophobic
PC10	Asp104	3.8	hydrophobic
PC10	Asn283	3.59	hydrophobic
PC10	Phe286	3.97	hydrophobic
PC10	Ala294	3.8	hydrophobic
PC10	Phe302	3.72	hydrophobic
PC11	Phe28	3.24	hydrophobic
PC11	Phe199	3.54	hydrophobic
PC11	Ala294	3.97	hydrophobic
PC12	Pro117	3.48	hydrophobic
PC12	Gln120	3.96	hydrophobic
PC12	Ile124	3.71	hydrophobic
PC12	Phe173	3.65	hydrophobic
PC12	Phe173	3.55	hydrophobic
PC12	Phe199	3.93	hydrophobic
PC12	Trp276	3.7	hydrophobic
PC12	Leu279	3.96	hydrophobic
PC12	Phe302	3.26	hydrophobic
PC12	Phe302	3.68	hydrophobic
PC12	Phe302	3.75	hydrophobic
PC13	Phe173	3.65	hydrophobic
PC14	Leu26	3.74	hydrophobic
PC14	Phe199	3.61	hydrophobic
PC14	Phe199	3.71	hydrophobic
PC14	Phe282	3.65	hydrophobic
PC14	Phe282	3.58	hydrophobic
PC14	Phe286	3.74	hydrophobic
PC14	Phe286	3.59	hydrophobic
PC14	Ala294	3.74	hydrophobic
PC14	His298	3.7	hydrophobic
PC14	Phe302	3.51	hydrophobic

Index	Residues	Distance (Å)	Type of interaction
PC14	Phe302	3.75	hydrophobic
PC15	Leu26	3.81	hydrophobic
PC15	Phe28	3.78	hydrophobic
PC15	Phe199	3.89	hydrophobic
PC15	Phe282	3.64	hydrophobic
PC15	Phe286	3.8	hydrophobic
PC15	Ala294	3.48	hydrophobic
PC15	His298	3.7	hydrophobic
PC15	Asn299	3.99	hydrophobic
PC16	Leu26	3.84	hydrophobic
PC16	Phe28	3.81	hydrophobic
PC16	Phe28	3.81	hydrophobic
PC16	Phe28	3.47	hydrophobic
PC16	Asp31	3.97	hydrophobic
PC16	Asp104	3.66	hydrophobic
PC16	Phe184	3.77	hydrophobic
PC16	Val197	3.86	hydrophobic
PC16	Phe199	3.58	hydrophobic
PC16	Phe199	3.76	hydrophobic
PC16	Phe286	3.63	hydrophobic
PC16	Phe286	3.73	hydrophobic
PC16	Ala294	3.35	hydrophobic
PC16	Phe302	3.5	hydrophobic
PC17	Phe28	3.69	hydrophobic
PC17	Phe28	3.6	hydrophobic
PC17	Phe28	3.85	hydrophobic
PC17	Phe28	3.56	hydrophobic
PC17	Phe199	3.98	hydrophobic
PC17	Phe199	3.52	hydrophobic
PC17	Phe282	3.59	hydrophobic
PC17	Phe286	3.71	hydrophobic
PC17	Phe286	3.81	hydrophobic
PC18	Phe28	3.96	hydrophobic
PC18	Val197	3.21	hydrophobic
PC18	Phe199	3.57	hydrophobic
PC18	Phe282	3.63	hydrophobic
PC18	Asn283	3.77	hydrophobic
PC18	Phe286	3.51	hydrophobic
PC18	Ala294	3.42	hydrophobic
PC18	Phe302	3.69	hydrophobic
PC18	Phe302	3.84	hydrophobic
PC19	Phe28	3.87	hydrophobic
PC19	Phe199	3.69	hydrophobic
PC19	Phe282	3.72	hydrophobic
PC19	Phe286	3.98	hydrophobic
PC19	Phe286	3.67	hydrophobic
PC19	Ala294	3.51	hydrophobic

Index	Residues	Distance (Å)	Type of interaction
PC19	His298	3.76	hydrophobic
PC20	Leu26	3.53	hydrophobic
PC20	Phe28	3.68	hydrophobic
PC20	Phe28	3.7	hydrophobic
PC20	Phe28	3.63	hydrophobic
PC20	Phe28	3.59	hydrophobic
PC20	Phe184	3.73	hydrophobic
PC20	Val197	3.32	hydrophobic
PC20	Phe199	3.53	hydrophobic
PC20	Phe282	3.66	hydrophobic
PC20	Asn283	3.57	hydrophobic
PC20	Phe286	3.67	hydrophobic
PC20	Ala294	3.92	hydrophobic
PC20	Phe302	3.61	hydrophobic
PC21	Phe28	3.59	hydrophobic
PC21	Phe28	3.57	hydrophobic
PC21	Phe28	3.56	hydrophobic
PC21	Phe28	3.75	hydrophobic
PC21	Asp104	3.75	hydrophobic
PC21	Val197	3.72	hydrophobic
PC21	Phe199	3.58	hydrophobic
PC21	Phe282	3.79	hydrophobic
PC21	Asn283	3.63	hydrophobic
PC21	Phe302	3.7	hydrophobic
PC21	Phe302	3.7	hydrophobic
PC22	Leu26	3.73	hydrophobic
PC22	Phe28	3.57	hydrophobic
PC22	Phe28	3.49	hydrophobic
PC22	Phe28	3.77	hydrophobic
PC22	Asp104	3.75	hydrophobic
PC22	Val197	3.64	hydrophobic
PC22	Phe199	3.43	hydrophobic
PC22	Phe199	3.72	hydrophobic
PC22	Phe282	3.77	hydrophobic
PC22	Asn283	3.71	hydrophobic
PC22	Phe286	3.04	hydrophobic
PC22	Phe286	3.52	hydrophobic
PC22	Phe302	3.57	hydrophobic
PC23	Leu26	3.38	hydrophobic
PC23	Phe28	3.65	hydrophobic
PC23	Phe28	3.61	hydrophobic
PC23	Tyr100	3.79	hydrophobic
PC23	Asp104	3.71	hydrophobic
PC23	Phe173	3.82	hydrophobic
PC23	Phe184	3.73	hydrophobic
PC23	Val197	3.48	hydrophobic
PC23	Phe282	3.69	hydrophobic

Index	Residues	Distance (Å)	Type of interaction
PC23	Asn283	3.74	hydrophobic
PC23	Phe302	3.63	hydrophobic
PC24	Leu26	3.62	hydrophobic
PC24	Phe28	3.95	hydrophobic
PC24	Phe28	3.9	hydrophobic
PC24	Asp104	3.82	hydrophobic
PC24	Phe184	3.81	hydrophobic
PC24	Val197	3.64	hydrophobic
PC24	Phe199	3.65	hydrophobic
PC24	Phe199	3.78	hydrophobic
PC25	Leu26	3.43	hydrophobic
PC25	Phe28	3.72	hydrophobic
PC25	Phe28	3.61	hydrophobic
PC25	Phe28	3.66	hydrophobic
PC25	Phe28	3.87	hydrophobic
PC25	Phe28	3.77	hydrophobic
PC25	Thr97	3.48	hydrophobic
PC25	Tyr100	3.48	hydrophobic
PC25	Asp104	3.67	hydrophobic
PC25	Pro117	3.99	hydrophobic
PC25	Val197	3.51	hydrophobic
PC25	Phe199	3.56	hydrophobic
PC25	Phe199	3.36	hydrophobic
PC25	Phe282	3.74	hydrophobic
PC25	Ala294	3.5	hydrophobic
PC25	Phe302	3.36	hydrophobic
PC25	Phe302	3.58	hydrophobic
PC25	Phe302	3.84	hydrophobic
PC26	Leu26	3.82	hydrophobic
PC26	Phe28	3.62	hydrophobic
PC26	Phe28	3.68	hydrophobic
PC26	Tyr100	3.71	hydrophobic
PC26	Asp104	3.72	hydrophobic
PC26	Pro117	3.93	hydrophobic
PC26	Phe173	3.91	hydrophobic
PC26	Phe184	3.82	hydrophobic
PC26	Val197	3.52	hydrophobic
PC26	Asn283	3.9	hydrophobic
PC26	Phe302	3.43	hydrophobic
PC27	Phe28	3.48	hydrophobic
PC27	Phe28	3.88	hydrophobic
PC27	Phe28	3.73	hydrophobic
PC27	Thr97	3.96	hydrophobic
PC27	Tyr100	3.68	hydrophobic
PC27	Tyr100	3.56	hydrophobic
PC27	Thr101	3.79	hydrophobic
PC27	Gln120	3.9	hydrophobic

Index	Residues	Distance (Å)	Type of interaction
PC27	Val197	3.64	hydrophobic
PC27	Phe199	3.73	hydrophobic
PC27	Ala294	3.8	hydrophobic
PC27	Phe302	3.49	hydrophobic
PC27	Phe302	3.72	hydrophobic
PC28	Thr280	3.54	hydrophobic
PC29	Leu26	3.56	hydrophobic
PC29	Leu26	2.74	hydrophobic
PC29	Phe28	3.68	hydrophobic
PC29	Asp104	3.98	hydrophobic
PC29	Phe199	3.63	hydrophobic
PC29	Phe199	3.91	hydrophobic
PC29	Asn283	3.61	hydrophobic
PC29	Phe286	3.19	hydrophobic
PC29	Phe286	3.77	hydrophobic
PC29	Phe302	3.79	hydrophobic
PC30	Leu26	3.48	hydrophobic
PC30	Phe28	3.82	hydrophobic
PC30	Phe28	3.39	hydrophobic
PC30	Phe28	3.75	hydrophobic
PC30	Thr97	3.55	hydrophobic
PC30	Tyr100	3.62	hydrophobic
PC30	Tyr100	3.62	hydrophobic
PC30	Pro117	3.94	hydrophobic
PC30	Val197	3.59	hydrophobic
PC30	Phe199	3.77	hydrophobic
PC30	Phe282	3.76	hydrophobic
PC30	Ala294	3.59	hydrophobic
PC30	Phe302	3.3	hydrophobic
PC30	Phe302	3.61	hydrophobic
PC30	Phe302	3.88	hydrophobic
PC31	Leu26	3.13	hydrophobic
PC31	Phe28	3.66	hydrophobic
PC31	Phe28	3.97	hydrophobic
PC31	Phe282	3.85	hydrophobic
PC31	Asn283	3.7	hydrophobic
PC31	Phe286	3.49	hydrophobic
PC31	Phe302	3.82	hydrophobic
PC31	Phe302	3.6	hydrophobic
PC32	Leu26	3.55	hydrophobic
PC32	Phe28	3.69	hydrophobic
PC32	Phe28	3.51	hydrophobic
PC32	Phe28	3.71	hydrophobic
PC32	Asp104	3.76	hydrophobic
PC32	Phe184	3.98	hydrophobic
PC32	Val197	3.71	hydrophobic
PC32	Phe199	3.22	hydrophobic

Index	Residues	Distance (Å)	Type of interaction
PC32	Phe199	3.89	hydrophobic
PC32	Asn283	3.73	hydrophobic
PC32	Phe286	3.81	hydrophobic
PC32	Phe302	3.62	hydrophobic
PC33	Leu26	3.47	hydrophobic
PC33	Phe28	3.92	hydrophobic
PC33	Phe28	3.73	hydrophobic
PC33	Phe28	3.68	hydrophobic
PC33	Phe28	3.86	hydrophobic
PC33	Phe28	3.67	hydrophobic
PC33	Asp104	3.67	hydrophobic
PC33	Val197	3.33	hydrophobic
PC33	Phe199	3.27	hydrophobic
PC33	Phe282	3.89	hydrophobic
PC33	Asn283	3.91	hydrophobic
PC33	Phe286	3.71	hydrophobic
PC33	Phe302	3.66	hydrophobic
PC34	Leu26	3.37	hydrophobic
PC34	Phe28	3.98	hydrophobic
PC34	Phe28	3.78	hydrophobic
PC34	Phe28	3.69	hydrophobic
PC34	Phe28	3.93	hydrophobic
PC34	Phe28	3.67	hydrophobic
PC34	Asp104	3.69	hydrophobic
PC34	Val197	3.39	hydrophobic
PC34	Phe199	3.32	hydrophobic
PC34	Phe199	3.9	hydrophobic
PC34	Phe282	3.89	hydrophobic
PC34	Asn283	3.82	hydrophobic
PC34	Phe286	3.87	hydrophobic
PC34	Phe302	3.62	hydrophobic
PC35	Leu26	3.35	hydrophobic
PC35	Phe28	3.82	hydrophobic
PC35	Phe28	3.86	hydrophobic
PC35	Phe28	3.66	hydrophobic
PC35	Phe28	3.87	hydrophobic
PC35	Phe28	3.71	hydrophobic
PC35	Thr97	3.65	hydrophobic
PC35	Tyr100	3.65	hydrophobic
PC35	Asp104	3.69	hydrophobic
PC35	Pro117	3.98	hydrophobic
PC35	Gln120	3.75	hydrophobic
PC35	Val197	3.46	hydrophobic
PC35	Phe199	3.57	hydrophobic
PC35	Phe199	3.48	hydrophobic
PC35	Phe282	3.65	hydrophobic
PC35	Ala294	3.63	hydrophobic

Index	Residues	Distance (Å)	Type of interaction
PC35	Phe302	3.45	hydrophobic
PC35	Phe302	3.57	hydrophobic
PC35	Phe302	3.8	hydrophobic
PC36	Leu26	3.38	hydrophobic
PC36	Phe28	3.79	hydrophobic
PC36	Phe28	3.65	hydrophobic
PC36	Phe28	3.92	hydrophobic
PC36	Phe28	3.66	hydrophobic
PC36	Asp104	3.68	hydrophobic
PC36	Phe184	3.99	hydrophobic
PC36	Val197	3.35	hydrophobic
PC36	Phe199	3.25	hydrophobic
PC36	Phe199	3.99	hydrophobic
PC36	Asn283	3.78	hydrophobic
PC36	Phe286	3.99	hydrophobic
PC36	Phe302	3.58	hydrophobic
PC37	Phe28	3.91	hydrophobic
PC37	Asp104	3.82	hydrophobic
PC37	Phe173	3.7	hydrophobic
PC37	Val197	3.46	hydrophobic
PC37	Phe199	3.72	hydrophobic
PC37	Phe199	4	hydrophobic
PC37	Leu215	3.73	hydrophobic
PC37	Phe282	3.32	hydrophobic
PC37	Phe286	3.2	hydrophobic
PC37	Phe286	3.95	hydrophobic
PC37	Ala294	3.23	hydrophobic
PC37	His298	3.76	hydrophobic
PC38	Leu26	3.46	hydrophobic
PC38	Phe28	3.85	hydrophobic
PC38	Phe28	3.41	hydrophobic
PC38	Phe28	3.8	hydrophobic
PC38	Thr97	3.44	hydrophobic
PC38	Tyr100	3.63	hydrophobic
PC38	Tyr100	3.61	hydrophobic
PC38	Pro117	3.85	hydrophobic
PC38	Val197	3.62	hydrophobic
PC38	Phe199	3.67	hydrophobic
PC38	Phe282	3.72	hydrophobic
PC38	Phe302	3.33	hydrophobic
PC38	Phe302	3.48	hydrophobic
PC38	Phe302	3.78	hydrophobic
PC39	Phe28	3.58	hydrophobic
PC39	Phe28	3.55	hydrophobic
PC39	Phe28	3.57	hydrophobic
PC39	Phe28	3.69	hydrophobic
PC39	Phe28	3.76	hydrophobic

Index	Residues	Distance (Å)	Type of interaction
PC39	Thr97	3.39	hydrophobic
PC39	Tyr100	3.21	hydrophobic
PC39	Asp104	3.65	hydrophobic
PC39	Phe184	3.88	hydrophobic
PC39	Val197	3.75	hydrophobic
PC39	Phe199	3.65	hydrophobic
PC39	Phe282	3.76	hydrophobic
PC39	His298	3.69	hydrophobic
PC39	Phe302	3.68	hydrophobic
PC39	Phe302	3.25	hydrophobic
PC39	Phe302	3.57	hydrophobic
PC39	Phe302	3.93	hydrophobic
PC41	Leu26	3.45	hydrophobic
PC41	Phe28	3.85	hydrophobic
PC41	Phe28	3.71	hydrophobic
PC41	Phe28	3.61	hydrophobic
PC41	Phe28	3.82	hydrophobic
PC41	Phe28	3.61	hydrophobic
PC41	Asp104	3.59	hydrophobic
PC41	Phe184	4	hydrophobic
PC41	Val197	3.29	hydrophobic
PC41	Phe199	3.41	hydrophobic
PC41	Phe199	3.95	hydrophobic
PC41	Phe282	3.87	hydrophobic
PC41	Asn283	3.6	hydrophobic
PC41	Phe286	3.09	hydrophobic
PC41	Phe286	3.74	hydrophobic
PC41	Phe302	3.52	hydrophobic
PC42	Leu26	3.62	hydrophobic
PC42	Tyr100	3.67	hydrophobic
PC42	Pro117	3.77	hydrophobic
PC42	Ile124	3.76	hydrophobic
PC42	Phe173	3.61	hydrophobic
PC42	Phe199	3.7	hydrophobic
PC42	Phe282	3.26	hydrophobic
PC42	Asn283	3.84	hydrophobic
PC42	Phe286	3.67	hydrophobic
PC42	Ala294	2.89	hydrophobic
PC42	His298	3.83	hydrophobic
PC42	Phe302	3.86	hydrophobic
PC43	Phe28	3.68	hydrophobic
PC43	Phe28	3.56	hydrophobic
PC43	Phe282	3.87	hydrophobic
PC43	Asn283	3.67	hydrophobic
PC43	Phe286	3.52	hydrophobic
PC43	Ala294	3.94	hydrophobic
PC43	Phe302	3.63	hydrophobic

**Index**	**Residues**	**Distance (Å)**	**Type of interaction**
PC43	Phe302	3.76	hydrophobic

Distance provided in Angstrom (Å).
