# Supplementary material for: Poria cocos compounds targeting neuropeptide Y1 receptor (Y1R) for weight management: A computational ligand- and structure-based study with molecular dynamics simulations identified beta-amyrin acetate as a putative Y1R inhibitor
Source: PLoS One. 2023 Jun 30;18(6):e0277873. doi: 10.1371/journal.pone.0277873 (PMC10313034; doi:10.1371/journal.pone.0277873)
Supplement: S1 Fig — Within each compound box, the following information were provided: PC index (top left), chemical formula (top right), PubChem CID (bottom center). (PDF) [file pone.0277873.s004.pdf]

S1 Fig. 2D structures of *Poria cocos* compounds with index (top left), molecular formula (top right), and PubChem CID.

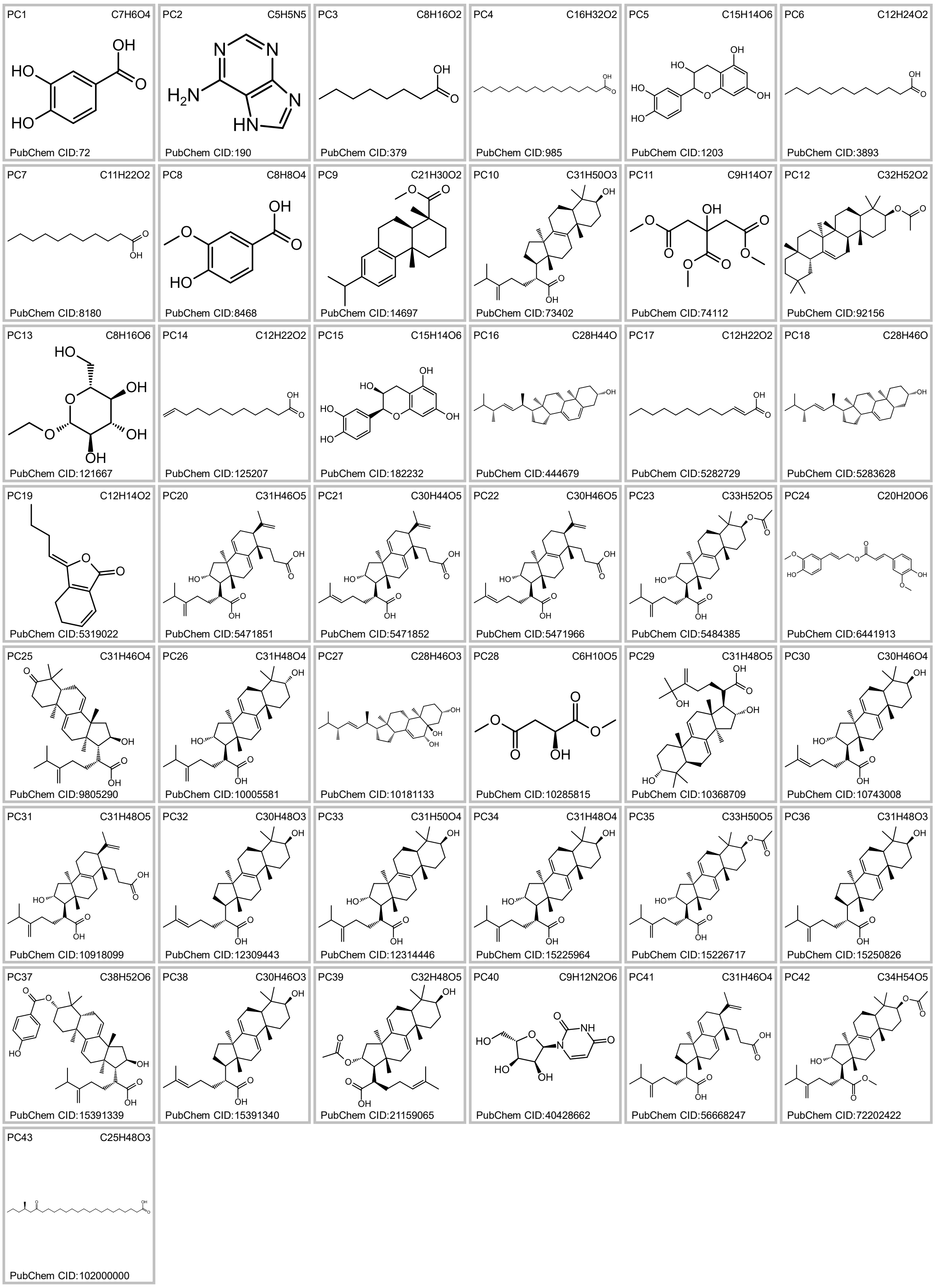

Within each compound box, the following information were provided: PC index (top left), chemical formula (top right), PubChem CID (bottom center).
