# Supplementary material for: Poria cocos compounds targeting neuropeptide Y1 receptor (Y1R) for weight management: A computational ligand- and structure-based study with molecular dynamics simulations identified beta-amyrin acetate as a putative Y1R inhibitor
Source: PLoS One. 2023 Jun 30;18(6):e0277873. doi: 10.1371/journal.pone.0277873 (PMC10313034; doi:10.1371/journal.pone.0277873)
Supplement: S2 Fig — For each continuous parameter, molecular weight was plotted in the x-axis against the parameter. Red line shows suggested thresholds for continuous parameters, where applicable. (PDF) [file pone.0277873.s005.pdf]

S2 Fig. Overview of ADMET properties of *Poria cocos* compounds based on pkCSM outputs.

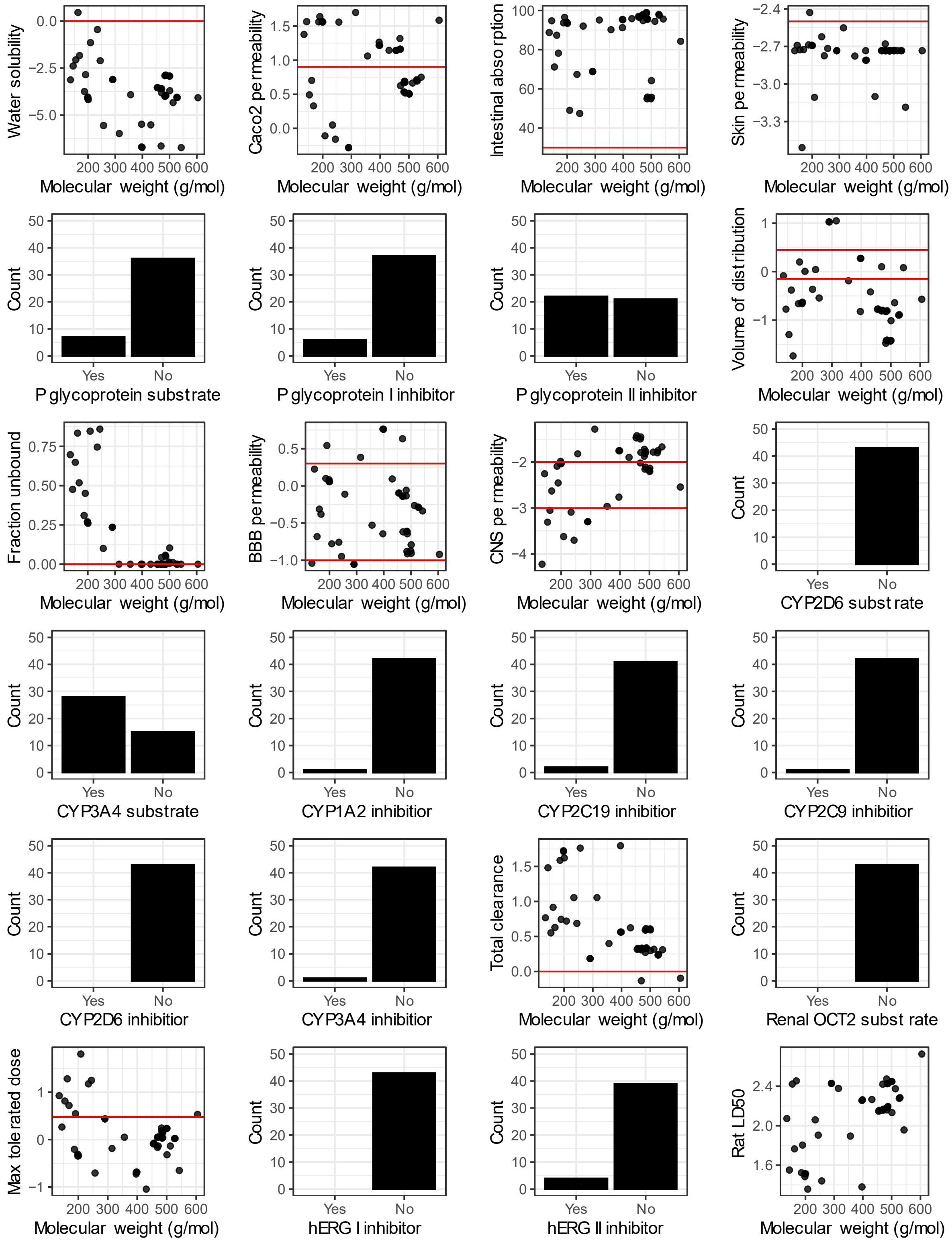

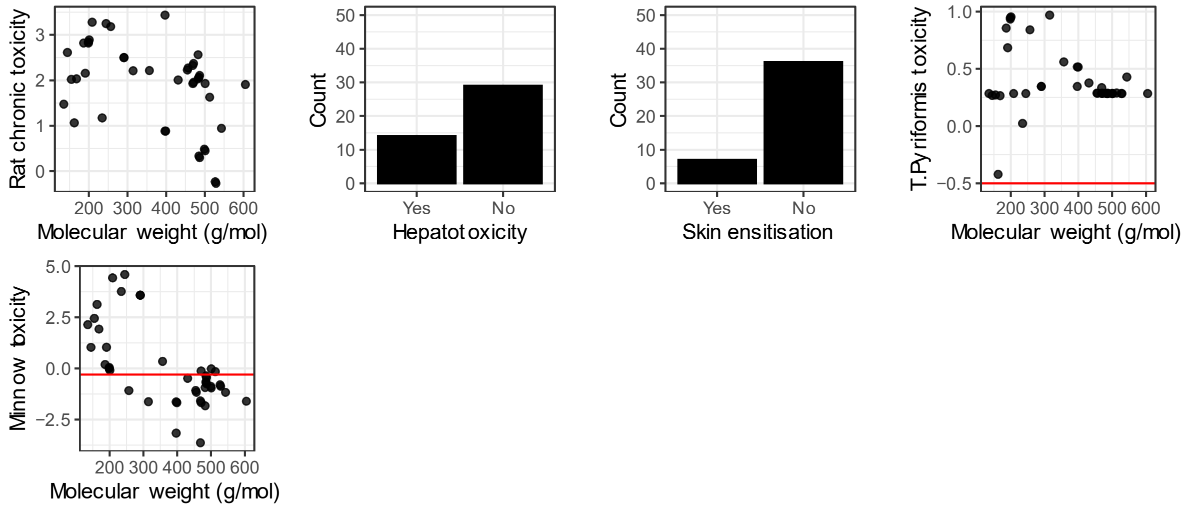

For each continuous parameter, molecular weight was plotted in the *x*-axis against the parameter. Red line shows suggested thresholds based on pkCSM guidelines, where applicable.
