# Supplementary material for: Poria cocos compounds targeting neuropeptide Y1 receptor (Y1R) for weight management: A computational ligand- and structure-based study with molecular dynamics simulations identified beta-amyrin acetate as a putative Y1R inhibitor
Source: PLoS One. 2023 Jun 30;18(6):e0277873. doi: 10.1371/journal.pone.0277873 (PMC10313034; doi:10.1371/journal.pone.0277873)
Supplement: S3 Fig — Ligand residue interactions in 3D and 2D views. (a) 3D diagram of 9AO superimposed in Y1R (green ribbons). (b) 2D diagram of 9AO (grey sticks) in its original co-crystallised form. (c) 2D diagram of 9AO (orange sticks) in blind docking. (d) 2D diagram of 9AO (yellow sticks) in focused docking. (PDF) [file pone.0277873.s006.pdf]

S3 Fig. Best binding poses of three molecules of co-crystalised known antagonist 9AO on Y_1R (PDB:5ZBQ).

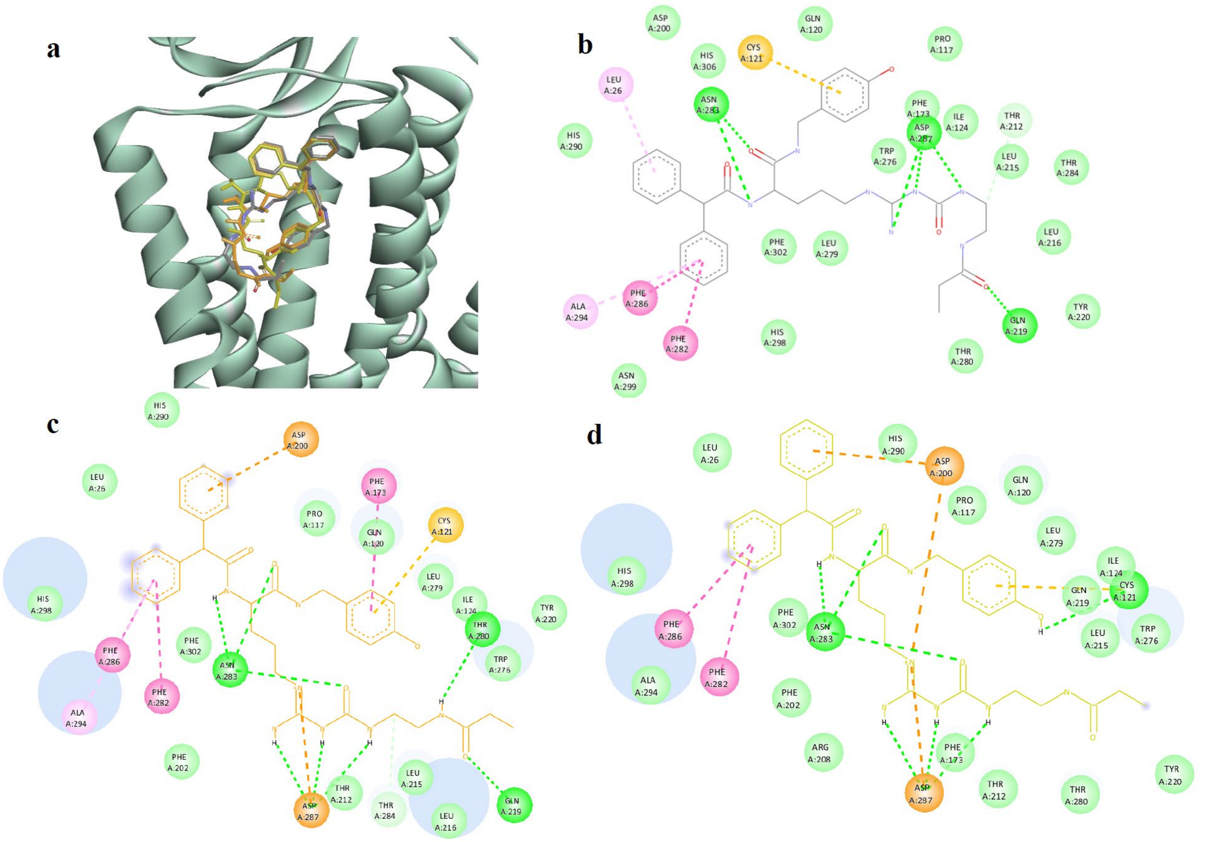

Ligand residue interactions in 3D and 2D views. (a) 3D diagram of 9AO superimposed in Y_1R (green ribbons). (b) 2D diagram of 9AO (grey sticks) in its original co-crystallised form. (c) 2D diagram of 9AO (orange sticks) in blind docking. (d) 2D diagram of 9AO (yellow sticks) in focused docking.
