# Supplementary material for: Poria cocos compounds targeting neuropeptide Y1 receptor (Y1R) for weight management: A computational ligand- and structure-based study with molecular dynamics simulations identified beta-amyrin acetate as a putative Y1R inhibitor
Source: PLoS One. 2023 Jun 30;18(6):e0277873. doi: 10.1371/journal.pone.0277873 (PMC10313034; doi:10.1371/journal.pone.0277873)
Supplement: S4 Fig — The colour of tiles represents an interaction detected within 4Å (green), and more than 4Å (white). The colour gradient represents the distance between the Phe residues and Poria cocos compounds, the closer the distance the darker the colour. (PDF) [file pone.0277873.s007.pdf]

S4 Fig. Interaction distance between *Poria cocos* compounds and residues of the hydrophobic phenylalanine (Phe) cluster in focused docking.

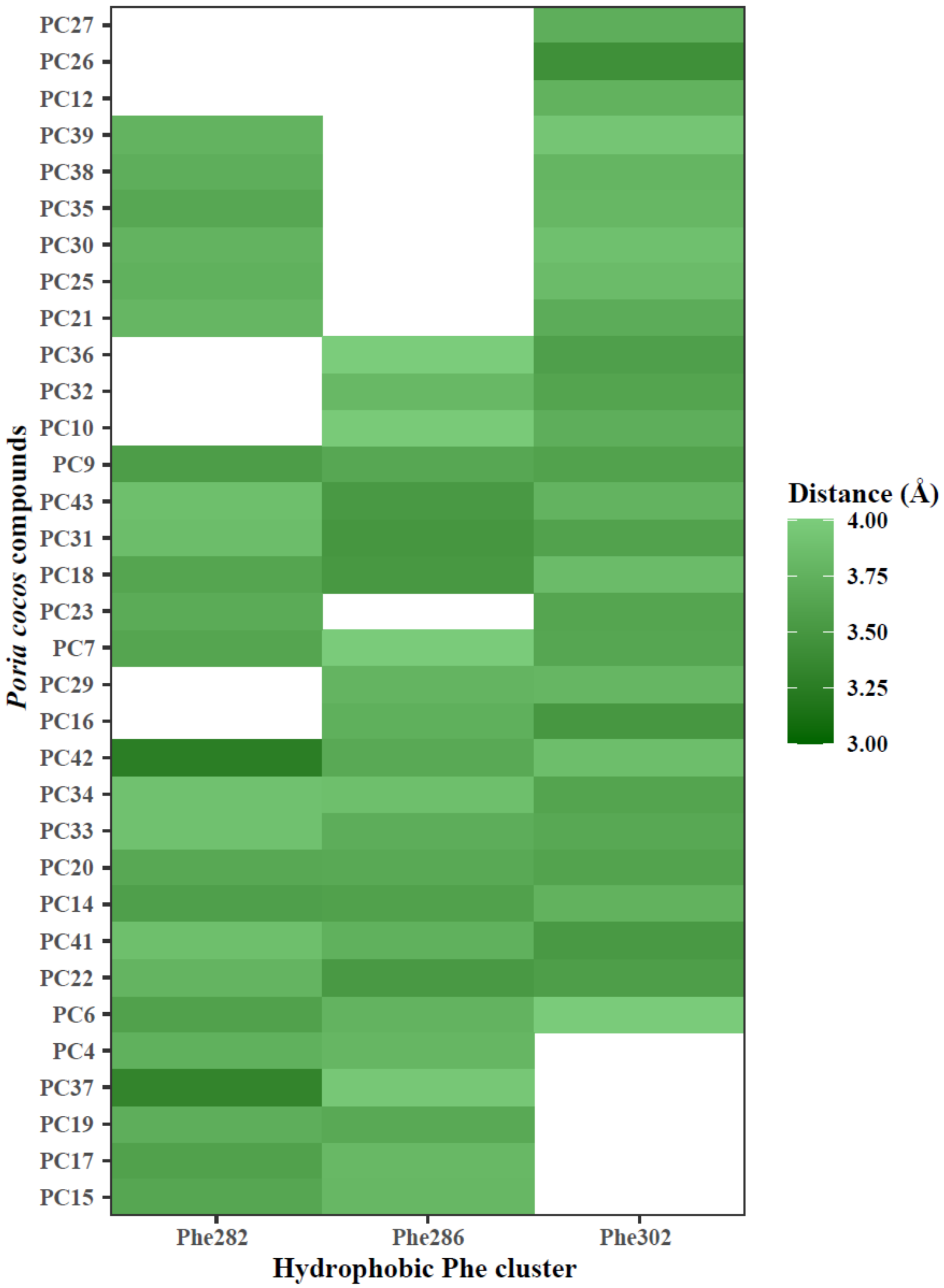

The colour of tiles represents an interaction detected within 4Å (green), and more than 4Å (white). The colour gradient represents the distance between the Phe residues and *Poria cocos* compounds, the closer the distance the darker the colour.
